# Magnetron Sputtered Lead Titanates Thin Films for Pyroelectric Applications: Part 1: Epitaxial Growth, Material Characterization

**DOI:** 10.3390/ma17010221

**Published:** 2023-12-30

**Authors:** Morteza Fathipour, Yanan Xu, Mukti Rana

**Affiliations:** 1 Division of Physics, Engineering, Mathematics and Computer Sciences & Optical Science Center for Applied Research, Delaware State University, Dover, DE 19901, USA; m.fathipour33@gmail.com; 2 Division of Physics, Engineering, Mathematics and Computer Sciences, Delaware State University, Dover, DE 19901, USA; yxu@desu.edu

**Keywords:** pyroelectric materials, perovskite lead titanate, calcium lead titanate, PCT, PZT, PLZT, PT

## Abstract

Pyroelectric materials, are those materials with the property that in the absence of any externally applied electric field, develop a built-in spontaneous polarization in their unit cell structure. They are regarded as ideal detector elements for infrared applications because they can provide fast response time and uniform sensitivity at room temperature over all wavelengths. Crystals of the perovskite Lead Titanate (PbTi$O_{3}$) family show pyroelectric characteristics and undergo structural phase transitions. They have a high Curie temperature (the temperature at which the material changes from the ferroelectric (polar) to the paraelectric (nonpolar) phase), high pyroelectric coefficient, high spontaneous polarization, low dielectric constant, and constitute important component materials not only useful for infrared detection, but also with vast applications in electronic, optic, and Micro-electromechanical systems (MEMS) devices. However, the preparation of large perfect, and pure single crystals of PbTi$O_{3}$ is challenging. Additionally, difficulties arise in the application of such bulk crystals in terms of connection to processing circuits, large size, and high voltages required for their operation. A number of thin film fabrication techniques have been proposed to overcome these inadequacies, among which, magnetron sputtering has demonstrated many potentials. By addressing these aspects, the review article aims to contribute to the understanding of the challenges in the field of pyroelectric materials, highlight potential solutions, and showcase the advancements and potentials of pyroelectric perovskite series including PbZrTiO_3_ (PZT), ${Pb}_{x}{Ca}_{1-x}$ (PZN-PT), etc. for which PbTi$O_{3}$ is the end member. The review is presented in two parts. Part 1 focuses on material aspects, including preparation methods using magnetron sputtering and material characterization. We take a tutorial approach to discuss the progress made in epitaxial growth of lead titanate-based ceramics prepared by magnetron sputtering and examine how processing conditions may affect the crystalline quality of the growing film by linking to the properties of the substrate/buffer layer, growth substrate temperature, and the oxygen partial pressure in the gas mixture. Careful control and optimization of these parameters are crucial for achieving high-quality thin films with desired structural and morphological characteristics.

## 1. Introduction

All matter absorbs and emits electromagnetic radiation with wavelengths (λ) ranging from 0.7 μm <λ< 1 mm, commonly referred to as the infrared (IR) spectrum. Both the absorption and emission spectra provide a wealth of valuable data useful for an abundance of applications. IR detectors facilitate access to this precious information via numerous tools designed for various commercial, industrial, medical, research, and military applications [1]. 

In some dielectrics, macroscopic polarization may be altered by such parameters as temperature, pressure, and electric field. This is manifested by pyro-, piezo-, and ferro-electricity, respectively. Thermal detectors convert the absorbed incident radiation, first into heat and then into polarization.

Pyroelectricity is often observed in perovskites (a cubic crystal with AB$O_{3}$ chemical formula belonging to a Pm-3m space group). When a perovskite-like material such as PbTi$O_{3}$, experiences a change in temperature, it may undergo a structural phase transition. This results in a rearrangement of its atomic configuration, which in turn induces a modification in its spontaneous polarization.

Increasing demand for lightweight, low-power, and low-cost IR detectors, as well as imaging heads that incorporate thin-film elements and can be integrated with silicon (Si) technology, have paved the way for the commercialization of uncooled pyro-electric IR (PIR) imaging systems, such as night vision instruments, fire rescue devices, biomedical thermography, security surveillance systems, and various other sensing devices capable of room temperature operation including different remote sensing devices and gas detectors as well as automotive, sailing, and aircraft cameras [2]. In particular, uncooled PIR detectors have received extensive attention, since they are lightweight and possess high sensitivity in a broad spectral bandwidth which is limited only by the ability of the sensor to absorb the incident radiation. Moreover, they can offer high-performance, room-temperature operation at an affordable price, operate in a wide temperature range, consume minuscule power, and provide relatively fast response [3].

## 2. Materials and Methods

Lead Titanate (PT) is an attractive material. It is a well-known ferroelectric with a relatively small dielectric constant (ε) along the polarization (or c-) axis, a large pyroelectric coefficient (p), and a large spontaneous polarization, which can be modified by external stimuli such as electric field, temperature, pressure, and radiation. Hence, it can provide a large voltage responsivity when utilized as an IR sensor. It has a high Curie temperature (T_c_), which makes it even more appealing for IR sensing applications. 

Still more interesting properties can be harvested by the partial replacement of Pb with another cation in a PT crystal to control or enhance such important material’s physical parameters such as p, ε, and Tc as well as the loss, electrical impedance, mechanical properties, etc. Thus, a wide range of dopants including Mn, Zr, Nb, and rare earth elements such as Ce, have been explored to achieve the above goals. For example, doping with certain elements such as Nd can enhance the pyroelectric coefficient of lead titanate. This means the material becomes more responsive to changes in temperature, making it suitable for pyroelectric applications. Besides that, doping with rare earth elements can lead to a shift in the Curie temperature of lead titanate while the Curie temperature is the point at which the material undergoes a phase transition, typically associated with changes in electrical and thermal properties. Rare earth element dopants provide a means to tailor this critical temperature [4]. Also, a handful of methods have been developed for the low-temperature synthesis of the above pyroelectric films. These include ion beam sputtering [5], electron beam co-evaporation [6], pulsed laser deposition [7], the sol-gel technique [8], magnetron sputtering [9], microwave-assisted sputtering [10], and chemical vapor deposition [11]. Among these techniques, magnetron sputtering enjoys several benefits over its counterparts, particularly in industrial applications, where achieving uniformly thick large-area films is a concern. These advantages include the deposition of high-purity films of metals, alloys, or compounds at high rates, excellent adhesion, unsurpassed step coverage, the ability to deposit films at relatively low temperatures, wonderful large area uniformity, and ease of automation at a lower cost than other techniques. The choice of deposition technique, including magnetron sputtering, depends on specific application requirements, materials, and other factors. Here is a comparison of magnetron sputtering with some alternative thin film deposition techniques in terms of efficiency, cost, and scalability [9]. When it comes to efficiency, magnetron sputtering can be used for a wide range of materials and is particularly effective for refractory materials and metals. The process enables precise control over film thickness and composition. Alternative Techniques such as electron beam co-evaporation are well-suited for producing multilayer or alloyed films with tailored properties [6]. Regarding the matter of cost, Magnetron sputtering systems can be relatively cost-effective, especially for small-scale applications. The equipment is commercially available, and the process can be scaled for production. For Alternative Techniques, such as Microwave-Assisted Sputtering, costs may be higher due to the addition of microwave components [10]. With respect to Scalability, Magnetron sputtering is highly scalable and is commonly used in both research and industrial settings. It can be adapted for large-scale production with multiple deposition sources. But Alternative Techniques, for instance, CVD can be scalable for large-area coatings, especially in certain industries such as semiconductor manufacturing and PVD such as thermal evaporation can be scaled for industrial production, but challenges may arise in maintaining uniformity over large substrates [11]. These criteria render the magnetron sputtering technique amenable to integrated circuit (IC) processing and encourage its utilization in both in situ and ex situ deposition (deposition followed by solid-state crystallization) of PT films with properties similar to those of their bulk counterparts. 

Magnetron sputtering is also a widely used thin-film deposition technique with several benefits when applied to the production and modification of bulk crystals [9]. This is because Magnetron sputtering provides excellent control over film thickness and uniformity. This is crucial for bulk crystals, ensuring a consistent and even coating across the crystal surface, which is important for various applications. Besides that, Magnetron sputtering allows for relatively high deposition rates, enabling efficient and timely coating of bulk crystals. This is advantageous for industrial-scale production.

Numerous researchers have reported on the preparation [12] and suitability of PT as a pyroelectric sensor [13,14]. Modified PT materials have shown interesting piezoelectric [15] and pyro-electric properties. Preparation and characterization of many modified versions of PT films including Calcium (Ca) or Calcium Lead Titanate (PCT) [16], Lanthanum (La)-modified PT (PLT) [17], and La modified Lead Zirconate Titanate (PLZT) [18] has been reported. Magnetron sputter deposition has also been used to synthesize layered perovskite films. These films have been utilized in a wide range of applications, not only as pyroelectric IR sensors [19,20,21] but also as high-value capacitors [22]. Lead Zirconate Titanate (PZT) possesses electro-optical behavior [23] and thin films of this material have been employed in ultrasonic transducers [24,25], thermistors [26], and optoelectronics [27]. Furthermore, thin films of ferroelectric PT have enabled the realization of nonvolatile random-access memory (NVRAMs) [28], certain kinds of microelectromechanical systems (MEMS) [29] as well as optical memories and displays [30]. PT-based alloys prepared by magnetron sputtering are very important for many applications [9,10], in which they can be effectively used to deposit thin films over bulk crystals by carefully controlling parameters such as surface preparation, adhesion promotion, substrate temperature, deposition rate, and in situ monitoring. These considerations are crucial for achieving high-quality thin films with the desired properties and ensuring compatibility with the underlying bulk crystal. But a complete review containing the growth and properties of these materials is missing. 

Understanding the epitaxial growth mechanisms is essential for controlling the crystalline quality, minimizing defects, and ensuring uniformity across the thin film. In this article, we investigate the growth of thin epitaxial films of the Lead Titanate family prepared by magnetron sputtering and outline the techniques commonly utilized for material and electrical characterization of such ceramics. Finally, we discuss how the growth parameters may affect the electrical characteristics of these pyroelectric films.

In Section 3 of this review article, we provide a brief overview of the physics of pyroelectricity. In Section 4, we discuss epitaxy and epitaxial growth of thin oxide films. This includes a brief overview of mechanisms governing the magnetron sputtering process of such films. Section 5 deals with parameters affecting the formation of PT films prepared by sputtering. We focus on the mechanisms affecting the growth kinetics as well as the thermodynamics of PT films by magnetron sputtering. Here, the issues involve the stability and stoichiometry control of the film. Section 5 handles the morphology of the films grown by magnetron sputtering.

## 3. Pyroelectricity in Materials

This section provides a concise and precise description of pyroelectricity and pyroelectric materials.

Pyroelectricity can be described as the ability of certain materials to generate a temporary voltage when they are heated up or cooled down. Such materials naturally collect charge at their surfaces and hence become electrically polarized. Consequently, they contain large internal electric fields and are characterized by a pyroelectric coefficient. Both polarization and the piezoelectric effect contribute to charge, as temperature is varied. Thus, p is modeled by describing the temperature derivative of the electric displacement vector (D) at constant electric field, E, and constant stress (σ) as:

(1) $$p={\left(\partial D/\partial T\right)}_{E,\sigma }$$

(2) $${\left(\frac{\partial D}{\partial T}\right)}_{E,\sigma }={\left(\frac{\partial D}{\partial T}\right)}_{E,s}+{\left(\frac{\partial D}{\partial T}\right)}_{E,T}{\left(\frac{\partial D}{\partial T}\right)}_{E,\sigma }$$

where s is the strain [1]. The first term on the right-hand side (RHS) of (2) is due to changes in polarization with temperature. For a pyroelectric material with constant dimensions, the term is called the “primary” pyroelectric effect. The second term on the (RHS) of (2) stems from dimensional changes of the pyroelectric material with temperature, while the material is kept under constant stress. This is because the piezoelectric effect results in an additional charge. For a ferroelectric material, the piezoelectric coefficient d is given as

(3) d=P+εE

where in (3) *P* is the polarization vector, which itself may be divided into a switchable part *Ps* and a non-switchable portion εE, i.e.,

(4) P=Ps+εE

Here, *ε* and *E* are the permittivity and the electric field of the ferroelectric material respectively. From (3) and (4), we may write,

(5) $$P={\left(\frac{\partial D}{\partial T}\right)}_{E,\sigma }={\left(\frac{\partial P_{s}}{\partial T}\right)}_{E,\sigma }{+E\left(\frac{\partial \varepsilon }{\partial T}\right)}_{E,\sigma }$$

where the first term on the (RHS) of Equation (5) is the primary pyroelectric coefficient (pp) at zero applied field and below material’s Tc, in which the pyroelectric material still behaves properly. The second term on the RHS of Equation (5) is significant near Tc, where dielectric permittivity becomes quite sensitive to temperature. From (5), assuming material dimensions remain constant, if E = 0, or dielectric permittivity is independent of temperature, the change in spontaneous polarization with temperature, denoted by pp [1]:

(6) $$p_{p}={\left(\frac{\partial P_{s}}{\partial T}\right)}_{E,\sigma }.$$

The secondary pyroelectric coefficient (*Ps*) is described as [1],

(7) $$p_{S}={\left(\frac{\partial D}{\partial S}\right)}_{E,T}{\left(\frac{\partial s}{\partial T}\right)}_{E,\sigma }{={\left(\frac{\partial D}{\partial \sigma }\right)}_{E,T}{\left(\frac{\partial \sigma }{\partial s}\right)}_{E,T}\left(\frac{\partial e}{\partial T}\right)}_{E,\sigma }=dc\alpha_{T},$$

where c is the elastic stiffness and $\alpha_{T}$ is the thermal expansion coefficient of the material. Both deformation and piezoelectricity contribute to *Ps*. A PIR may be designed to operate below Tc, above Tc, or switch between these two states.

## 4. Epitaxial Growth of Lead Titanates by Magnetron Sputtering

Sputtering has been extensively used in IC manufacturing not only because it is an economical process, but also because it provides good uniformity, step coverage, and high deposition rates [31]. Furthermore, proper choice of substrates enables this technique to support in situ epitaxial growth of many crystalline oxides including, perovskite PT with controlled growth orientation and excellent electrical properties.

### 4.1. Epitaxy and the Parameters Influencing the Epitaxial Growth

Epitaxy refers to the process through which a single crystal film is grown on top of another single crystal (substrate) of either the same (homo-epitaxy) or of a different (hetero-epitaxy) material. The achievement of epitaxial growth in the magnetron sputtering process involves a complex interplay of lattice mismatch, which reduces the formation of defects such as dislocations and promotes the continuation of the crystal lattice from the substrate to the growing film, interfacial energy in which low interfacial energy is desirable for epitaxial growth, as it encourages the alignment of crystal structures at the interface, and chemical bonding that ensures good adhesion and facilitates the transfer of crystallographic information from the substrate to the growing film [31]. By controlling the nature of nucleation and growth, it is feasible to achieve thin films with good crystalline quality.

**Figure 1:**
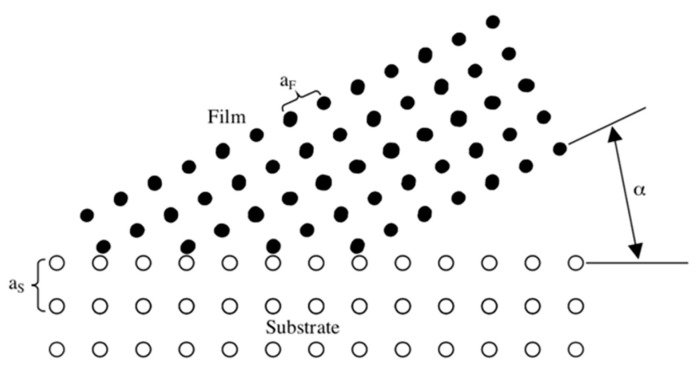
Schematic representation of the epitaxy by tilting the growth direction of the film relative to the substrate plane [36]. “Reproduced with permission from Copyright Clearance Center on behalf of Smith, D. L., Thin-film deposition: principles and practice; McGraw-Hill: New York, USA, 1995”.

A single-crystal film without any microstructure is ideal for pyroelectric applications. However, heteroepitaxial PT thin films are generally composed of microstructures, such as multidomain structures. The origin of the microstructure is related to the lattice mismatch, either thermal or mechanical, between the PT film and the substrate crystal [32,33] and/or the growth conditions [34] and is essentially governed by the initial stage of the film growth. Three mechanisms are responsible for the formation of the microstructure. These are: (a) layer-by-layer or lateral growth (Frank–Van der Merwe), (b) 3-D nucleation and island growth (Volmer–Weber), and (c) mixed mode (Stranski–Krastanov or SK) growth. Among these growth modes, the desired one is 2-D lateral growth, where one or a few layers of atoms form in a continuous, defect-free way through the coalescence of neighboring monolayer islands to minimize interfacial energy. This may be achieved by the deposition of perovskite on a miscut substrate under a step-flow growth mode [35]. The use of a miscut substrate is a means to avoid large misfit strain, at least along the interface. As seen in Figure 1, the growth direction of the film is at an angle α to the substrate normal. Lattice mismatch is defined as the relative difference between the in-plane lattice constants.

In the step-flow growth mode, the movement of adatoms (atoms or molecules on the surface) along terraces is influenced by growth anisotropy, and this anisotropy strongly affects the shape of terrace edges, making them elongated or differently shaped in different crystallographic directions. When the total pressure ${\;P}_{T}$ (${=P}_{O_{2}}$ + $P_{{Ar}_{2}})$ is above a critical value (P_cr_), the adatoms cannot diffuse far enough on the terrace to create a complete layer. Instead, a two-dimensional nucleation and island growth mechanism is initiated. On the other hand, when ${\;P}_{T}$ < P_cr_, the adatoms can diffuse on the terrace far enough to facilitate layer-by-layer growth. Under the given preparation conditions, including the miscut angle and the oxygen partial pressure, the layer growth mode will be stable in a high-temperature zone. Generally, the miscut angle lies within ±0.5° for the conventional single crystals.

Layer-by-layer growth mode requires stringent control of chemical composition including low $O_{2}$ pressure and low deposition rate, but leads to a smooth surface, with an atomic scale uniformity, which assures the prevalence of the epitaxial growth. The growth mechanism is governed by the surface mobility of the adatoms on the substrate during the film growth. 

**Figure 2:**
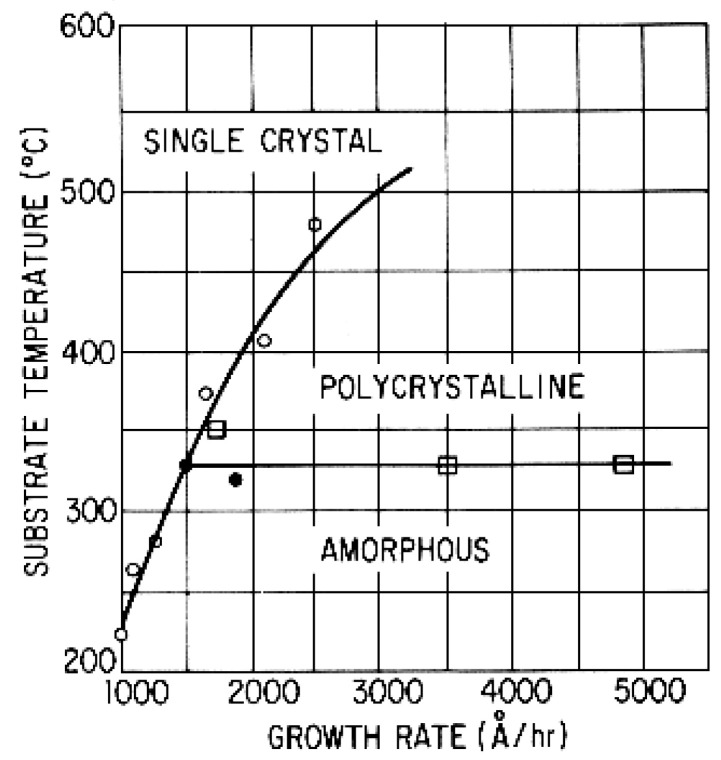
Evolution of crystal phases as a function of on Ge (111) substrate [37]. Copyright © Greene J.E. Epitaxial crystal growth by sputter deposition: Applications to semiconductors. Part 2. Critical Reviews in Solid State and Material Sciences 1983, 11(3), pp: 191, Figure 23, https://doi.org/10.1080/01611598308244063, reprinted by permission of Informa UK Limited, trading as Taylor & Francis Group, www.tandfonline.com on behalf of Greene J.E. Epitaxial crystal growth by sputter deposition: Applications to semiconductors. Part 2. Critical Reviews in Solid State and Material Sciences 1983, 11(3), pp: 191. https://doi.org/10.1080/01611598308244063.

In traditional vapor phase epitaxy, molecules arriving at the substrate gain most of their energies in the form of thermal energy from the environment and/or the substrate. This requires growth to be carried out at an elevated substrate temperature (*Ts*). For most materials, *Ts* is close to the melting point (Tm). A rule of thumb is *Ts* = (0.7–0.8) Tm. Hence epitaxial growth temperature, Tepi (=*Ts*) is usually large. Note that in general, Tepi > Tcrys, where Tcrys is the crystallization temperature. This is because Tepi is governed by the non-thermal equilibrium process in the vapor-to-solid phase transition, during the vapor phase epitaxial growth of the film, while Tcrys is determined by the thermal equilibrium process and governed by the crystallization phenomena for the film itself, analogous to what is encountered in the sintering of ceramics. For PCT films with Tm = 1975 °C, this gives *Ts* = Tepi ≈ 1400 °C. The evolution of crystal phases as a function of substrate temperature and growth rate is depicted in Figure 2, for the epitaxy of Ge on Ge (111) substrate. It is seen that below a certain *Ts*, only an amorphous phase is expected. Furthermore, for a given growth rate, the polycrystalline (poly) or the amorphous phase prevails at lower *Ts*.

Nevertheless, processing of PT films at high temperatures is quite undesirable especially for IC processing. Fortunately, in the sputtering process, species arriving at the substrate gain most of their energies in the form of kinetic energy, from the electric field, while accelerating in the plasma sheath [38,39]. This aids sputtering to support epitaxial growth of metal oxides at substrate temperatures as low as (0.2–0.3) Tm, which is quite practical and amenable to IC processing.

(a) Thin films of the single crystalline phase are deposited epitaxially on appropriate single crystal substrates at *Ts* = Tepi. The epitaxial temperature depends on both the film and the substrate material. In the case of magnetron sputtering of PT, Tepi~575 °C.
(b) Thin films of the polycrystal phase are deposited at *Ts* > Tcrys.
(c) The amorphous thin films deposited on the single crystal substrates also convert into the single-crystalline phase after the post-annealing at a temperature Tpan > Tepi, by solid phase epitaxy as discussed in Section 4.2. In the case of ex situ magnetron sputtering of PT, Tpan~750 °C.

Mechanisms (b) and (c) are both undesirable since they cause defects in the growing film, including twins and/or stacking faults, and increase the film’s surface roughness. The excellent piezo- and pyro-electric properties of bulk ferroelectrics are degraded when the ferroelectric is processed into thin films. This is because the grain boundaries are formed randomly [40,41]. Thus, controlling the nature of nucleation and growth are the two important factors in achieving a good crystalline thin film. 

### 4.2. Magnetron Sputtering and the Parameters Influencing the Sputtering Deposition Process

Sputtering refers to a momentum transfer process, by which energetic species, mainly positive ions, present in an inert plasma (a collection of the positive ions of an inert gas and free electrons) of a magnetically enhanced glow discharge, bombard and eject species from a target (source). The ejected particles then travel through the plasma, colliding with plasma species, before being deposited on a substrate. Film morphology is affected by (i) The flux, (ii) the plasma power, and (iii) the average incidence angle of bombarding ions.

Non-conductive materials may be sputter-deposited by supplying radio frequency (RF) power to the target, while for the sputtering of conductive materials either RF or DC power can be utilized. In the synthesis of PT thin films, oxygen and an inert gas, usually ${Ar}_{2}$, is employed. However, the undesirable reaction of the reactive gas with the target material may result in the nonlinear behavior of the deposition parameters as a function of the reactive gas flow.

Commonly, it is desired that deposition processes are carried out at low pressures (<1 Pa). However, reducing processing pressure increases the electron mean free path (MFP) allowing for the production of ions farther away from the target. Then, primary electrons are either lost to the chamber walls or directly access the anode without ionizing gas molecules, effectively reducing overall ionization efficiency. Consequently, at pressures lower than 1 Pa, the plasma extinguishes. To enhance sputter efficiency, a magnetic field is utilized. This process is referred to as magnetron sputtering. The operation relies on implementing a specially shaped magnetic field in a diode-sputtering target. In this manner, electrons are trapped in a localized helical path region close to the cathode, which increases their residence time in the plasma and vastly boosts their ionizing collision probability with plasma particles. This greater ionization efficiency leads to the enhancement of the ionic current density arriving at the target. Thus, the sputtering rate of the target is proportionally increased [40,42]. 

The relation between the maximum deposition rate $\left(v_{max}\right)$ and substrate temperature ($T_{s}$) for epitaxial film is given by [43]:

(8) $$v_{max}=C\;exp\left(E_{a}/RT_{s}\right),$$

where C is a constant, R is the ideal gas constant, and $E_{a}$ is the activation energy for epitaxial film formation [44,45].

Due to the linkage between its processing variables involved, differences in sputter yields of target elements, the effects of re-sputtering on the depositing film, differences in collisional cross sections of sputtered materials and plasma species (caused by differences in atomic masses of constituent target elements), sputtering from compound targets, such as those employed for deposition of PT family members, involve a complex transport process. Important issues include control of the film stoichiometry and crystallinity, the choice of target and substrate as well as the partial pressure and total pressure processing gasses throughout the film growth. To address these issues, we discuss below the processes occurring (1) at the target (2) between the target and the substrate, and (3) at the substrate, separately [44].

1. Processes occurring at the source (target)

For the preparation of the stable thin films of PT family members, composite targets are generally selected. Other considerations important in selecting a target material include: target purity, sputtering efficiency, and stoichiometry of the target [45]. 

At the target end, three processes, stemming from differences in (a) sputtering rates, (b) the mean free path and/or (c) the vapor pressures of the constituent target species, contribute to changes in film stoichiometry. 

For a multi-component target, at least at first glance, it would seem natural that the differences in the sputtering yield of the target species would cause the target to become depleted first from the species with the highest sputtering yield, then from the next element with the second highest sputtering yield and so on, a process which may raise concerns regarding the compositional uniformity of the growing film. Fortunately, this is automatically prevented in practice. This is because, once the target is depleted from the particles with the highest sputtering yield, the flux of this species decreases with time until a steady state is reached, where the film stoichiometry becomes the same as that of the target’s bulk composition. Thus, when any deposition parameter (such as pressure, process gas ratio, or the plasma power) is changed, or whenever a new target is used, a lengthy period (at least several hours) of pre-sputtering should be exercised to ensure deposition of a film with a bulk stoichiometric composition of the target material. In cases where the gas species modify the effective target surface area during the sputtering process, say by engraving it, the above argument may not hold. In RF sputtering of PT, the adoption of PbTi$O_{3}$ powder targets has been a prevalent practice, although ceramic targets have also been used [46].

Due to the high vapor pressure of lead (melting point = 327.5 °C), Pb-containing targets further complicate the stoichiometry control of the depositing material. Thus, as a common practice, an extra fraction of PbO has been incorporated into the powder composition of the PCT to compensate for Pb deficiency in the deposited film [47,48,49].

2. Processes between the target and the substrate

Once species are sputtered off the target, they travel through plasma and arrive at the target after accelerating through the plasma sheath. To avoid re-sputtering by negative oxygen ions (discussed in the next subsection), it is a common practice to choose a substrate-target distance, $D_{st}$, much larger than the mean free path of the target species, λ, given by [47]:

(9) $$\lambda =\frac{RT}{\pi {d_{a}}^{2}N_{a}P\sqrt{2}}$$

where, R is the ideal gas constant, λ represents the mean free path of the species, $d_{a}$ is the atomic diameter, P is the deposition pressure and $N_{a}$ is the Avogadro’s number. At high chamber pressures, sputtered species become molecular in nature and may encounter one or more collisions during their journey to the target, thus the deposition rate curtails. For P=27 Pa (189 mTorr), the mean free path of PT molecules, λ is approximately 1 mm [47]. At low chamber pressures, ($\lambda /D_{st}$) becomes large and species contributing to the film growth are atomic in nature. 

Reducing $D_{st}$, increases the deposition rate, but also adds to the film’s non-uniformity. Furthermore, energetic (neutral) oxygen atoms coming off of the oxide target can re-sputter the lighter elements in the growing film and deplete such elements from the film.

Numerous film characteristics such as film composition, microstructure, and thus film’s electrical properties are controlled by the complex plasma-surface interaction and material transport through the plasma [50].

To overcome difficulties associated with the high vapor pressure of PbO, a multi-source target has also been used. Here, stoichiometry and preferential growth (see below) of the films are mainly governed by (a) reproducible formation of an oxide layer on the target surface, (b) formation of a stable oxide during the transport of sputtered species toward the substrate, and (c) the nucleation and growth of the film on the substrate surface. PT and La- modified PT films have been prepared by using a multi-target DC sputtering system [47]. One cathode consisted of either Pb and La and the other of Ti. This design allows for the independent control of the vapor pressure of each element using an independent DC voltage. Oxides deposited in an inert gas, tend to become oxygen deficient. Hence, sputtering was carried out in an ${Ar}_{2}/O_{2}$ gas mixture. Films were deposited on (0001) sapphire. The crystallinity of the resulting epitaxial films is improved when (Pb/Ti) in the target approaches 1.1. Re-evaporation of Pb from the substrate is apparently more remarkable in La-modified PT film than in PT.

3. Processes occurring at the depositing film

Loss of certain species in the depositing film, referred to as preferential sputtering, is one of the major issues at the depositing film. Several sources, including (a) re-sputtering, (b) differences in sticking coefficients of sputtered species, (c) surface diffusion, and (d) re-evaporation of one or more of the film constituents, contribute to this phenomenon. Re-sputtering of the growing film by negative ions and reflected neutrals results in the formation of various features such as pits, ripples, cones, and craters on the film surface [51] examined the particles incident on the PZT thin films during sputtering and their energy distribution, under the conditions of 3.3 Pa, ${Ar}_{2}$ sputtering gas, and 150 W. They found that most of the particles incident on the substrates are ${Ar}^{+}$ ions whose energy is 23 eV. They argue that $O^{-}$ ions energy is much higher, in the range of 100 to 400 eV, since these ions are accelerated by the electric field in the plasma sheath. Thus, $O^{-}$ ions can jeopardize the film uniformity, even though they are much smaller in number, approximately (1/150) times the number of ${Ar}^{+}$ ions. 

Grace et al. [52] discussed possible mechanisms of negative ion formation in the magnetron environment and suggested that molecular oxygen can bring about re-sputtering primarily by forming $O_{2}^{+}$, which collides with the target to produce energetic negative oxygen ions. In contrast, ozone may form negative ions by electron impact in the dark space above the target, giving rise to lower-energy negative ions, which can then traverse the plasma without getting neutralized but can be stopped with a small negative bias applied to the target. With no added oxidant, negative oxygen ions from the target may dominate the re-sputtering phenomenon. Re-sputtering can be minimized by thermalizing (reducing the kinetic energy of) the energetic species in the plasma either by sputtering in a high-pressure environment or by off-axis sputtering. Sputtering from chamber walls, especially by reflected primary ions, may modify film composition. Also, gaseous species (working or residual) may be incorporated in the film either by adsorption of reactive gases (e.g., oxygen as encountered in oxidation) or by implantation of reflected primary ions into the growing film [40,53]. 

During the RF sputtering, a negative DC bias voltage may be directly coupled to the substrate through an inductor. This technique is referred to as bias sputtering. The inductor serves to block spurious AC signals. Then, the DC bias voltage enhances the kinetic energy of the depositing species, thus facilitating their chemical reaction and assisting the crystallization of low-energy positive ions. Furthermore, it discourages energetic negative oxygen ions bombardment of the growing film. Researchers [54,55] reported that in the presence of a negative bias voltage, sharp lines appear near 2θ=32°–33°, in the X-ray diffraction pattern of the deposited film. Furthermore, as the bias voltage is increased, loss of tangent (tan⁡δ) decreased while the relative dielectric constant ($\varepsilon_{r}$) increased. Also, the film surface deposited under the bias voltage possessed a much smoother appearance than that of the film deposited in the absence of the bias voltage. This implies that the surface morphology of the growing film can be modified during sputtering. 

A technique commonly referred to as “microwave-assisted sputtering” was proposed by Okumara et al. [10]. They exploited the microwave power at a frequency of 2.4 GHz in conjunction with the plasma in an RF magnetron sputtering system to trigger the plasma thus promoting chemical reactions of the sputtered species. Their analysis reveals that the crystalline and dielectric properties of the PT films prepared by this technique are improved in comparison to those prepared by the conventional sputtering method.

### 4.3. Parameters Affecting Epitaxial Growth of Perovskite Lead Titanates by Magnetron Sputtering

Traditionally, one of the following two routes has been utilized for the preparation of PT films by magnetron sputtering:

1. growth using in situ substrate heating, i.e., the deposition of the oxide film at a sufficiently high substrate temperature to allow in situ formation of perovskite phases [19,56,57].
2. solid-state crystallization, i.e., low-temperature deposition of amorphous film followed by solid-state crystallization using post-annealing in $O_{2}$ [50]. These two routes are discussed below.

#### 4.3.1. Growth Kinetics Using In Situ Substrate Heating

In situ growth of perovskite films is a desirable route, since it simplifies processing steps. As explained below, the growth mechanisms leading to the formation of PT films deposited by sputtering are governed by the degree of the re-evaporation of PbO from the film during the growth. The degree of re-evaporation, itself, depends on the lead content of the film.

1. Dependence of the PT film’s atomic ratio on the target’s lead content;

Jaber et al. [56,58] employed RF magnetron sputtering with in situ heating to prepare Stoichiometric, polycrystalline, transparent, and crack-free PT thin films on (0001) sapphire substrates. A summary of the growth parameters is given in Table 1.

**Figure 3:**
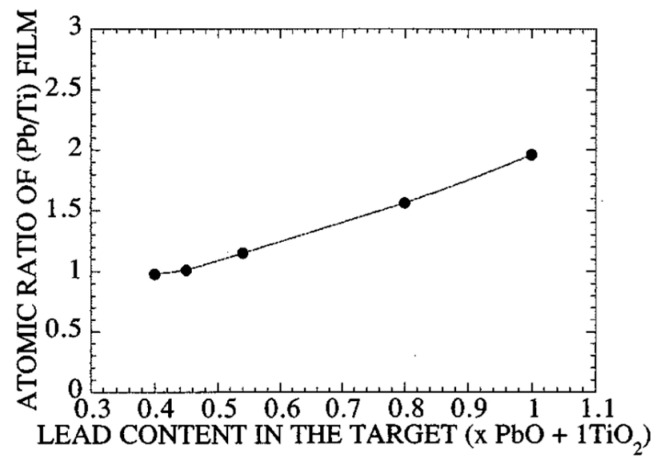
Atomic ratio (Pb/Ti) in the PT films produced by magnetron sputtering with no in-situ heating is used for the film growth. Oxide targets was composed of $xPbO+1TiO_{2}$, with 0.4<x<1.0 [56]. “Reproduced from Jaber, B.; Remiens, D; Thierry, B. Substrate temperature and target composition effects on PbTiO_3_ thin films produced in situ by sputtering. Journal of Applied Physics, 1996, 79(2), p.1182–1184. https://doi.org/10.1063/1.360903, with the permission of AIP Publishing”.

Jaber et al. [56,58] analyzed the composition of the films obtained with different targets composed of 1PbO + xTi$O_{2}$(0.4<x<1.0). The results are shown in Figure 3, for the case where no substrate heating was utilized. The films contained a high amount of excess Pb content for the target with x=1. Excess Pb (Pb/Ti=1.15) was detected even for the target with x=0.54. Assuming ${Ar}^{+}$ ion projectiles, sputtering threshold energies for commonly used materials are listed [70].

**Figure 4:**
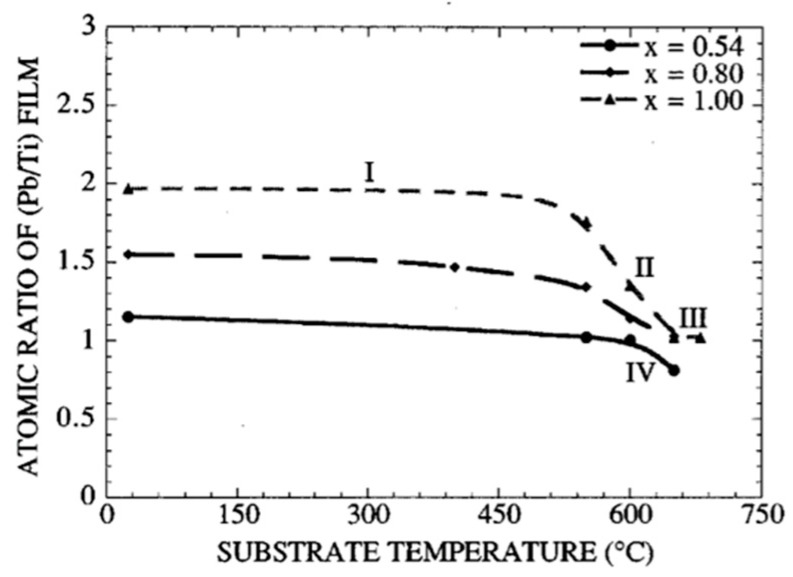
(Pb/Ti) ratio of films obtained at various substrate temperatures and target compositions [56]. “Reproduced from Jaber, B.; Remiens, D; Thierry, B. Substrate temperature and target composition effects on PbTiO_3_ thin films produced in situ by sputtering. Journal of Applied Physics, 1996, 79(2), pp. 1182–1184. https://doi.org/10.1063/1.360903, with the permission of AIP Publishing”.

2. Dependence of the film’s atomic ratio on the substrate’s growth temperature;

Figure 4 shows (Pb/Ti) of the film vs. *Ts*, for three targets with x=0.54, 0.8, and 1.0. Four characteristic regions are observed. In region I, (0<Ts<500 °C), the (Pb/Ti) ratio of the film remains nearly constant for any of the three values of lead content. Evidently, the re-evaporation of Pb species is negligible at these lower temperatures. However, in region II, the (Pb/Ti) decreases rapidly, indicating the re-evaporation of lead components from the films is becoming predominant. Such a region is observed for all three targets.

The higher (Pb/Ti) the ratio of the films (e.g., those resulting from targets with x=0.8 and x=1), the higher the degree of re-evaporation of the Pb species. In region III, the film’s (Pb/Ti) ratio approaches a constant value corresponding to the stoichiometric PT composition. Such a region is observed independent of the lead oxide content of the target but at different temperature zones. Evidently, in this region, the Pb species directly react with Ti species to yield stoichiometric PbTi$O_{3}$, which is incorporated into the film to form the perovskite film. The excess lead component, not incorporated in perovskite film, is re-evaporated. Region IV, is observed only for the films with small excess lead content (i.e., those resulting from a target with x=0.54). 

At high temperatures, the re-evaporation rate of PbO during the growth increases, such that zone III totally vanishes. Thus, the resultant film is often Pb deficient. Therefore, the sputtered lead flux (i.e., the lead content in the target), must be increased in order to obtain a stoichiometric film. For the same temperature range, region IV is not observed for targets with x=0.8 and x=1, because a higher growth temperature is necessary to favor sufficient re-evaporation of PbO.

In other words, with an appropriate combination of the Ts and x, single-phase perovskite films can be prepared at low temperatures. Films have the correct Pb stoichiometry for x=0.8 when 600<Ts<650 °C and for x=1, when 650<Ts<680 °C, while for For x=0.54, with $0.54\;PbO+1TiO_{2}$ and P=100 mT [58] the film’s (Pb/Ti) remains constant at a value close to one, in the temperature range 550 °C<Ts<600 °C. This self-stabilization effect, i.e., the saturation of the film’s lead content at the stoichiometric composition for a given growth temperature range, which has also been observed for the lead titano-zirconate films, is attributed to the preferential re-evaporation of the volatile lead oxide (PbO) species from the perovskite film during the deposition process. At higher growth temperatures, the re-evaporated lead flux must increase in order to obtain a correct lead stoichiometry of (Pb/Ti) =1, For x=0.54 and P=100 mT, analysis of X-ray diffraction patterns of the poly films reveals that the films grown at 600 °C assume a preferred (111) orientation, while the poly films prepared at Ts=550 °C *Ts* do not find any preferential orientation [57]. This is attributed to the higher mobilities of the sputtered species at higher temperatures. The incident Pb flux reduces the growth temperature. The growth mechanism of PT is governed by the degree of re-evaporation of Pb species, which itself depends on the lead content of the film: a competition exists between the re-evaporation of PbO during the formation and growth of PT by the reaction of PbO with Ti$O_{2}$ and is favored for Ts>550. Two parameters affect this competition: the substrate temperature and the sputtered lead flux arriving at the substrate. The perovskite phase formation increases with x. This is the consequence of a competition between the Pb species’ arrival at the substrate and their re-evaporation from the film during the growth. Then, the excess Pb, not incorporated in the perovskite structure, is re-evaporated and the stoichiometric films are obtained with this self-control mechanism. However, a limit exists; for x<0.54, it is impossible to grow in situ perovskite films, whatever the substrate temperature (the films become Pb deficient). 

At Ts=550 °C, superior epitaxial quality is achieved for x=0.54. Also, E. Dogheche et al. [58] obtained dense and transparent PT films with a thickness of 280 nm having relatively flat surfaces and no apparent grain boundaries thin films at Ts=550 °C on (100) SrTi$O_{2}$ substrate.

**Figure 5:**
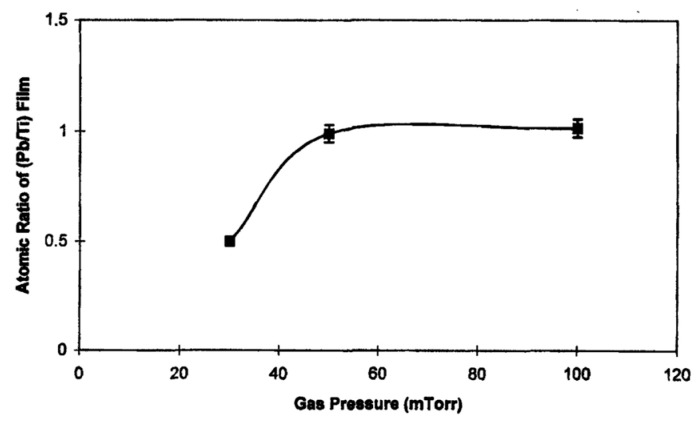
(Pb/Ti) ratio for the PT films prepared at *Ts* = 550 °C using a target with x=0.54 at various gas pressures [58]. Copyright © Jaber, B.; Dogheche, E.; Rèmiens, D.; Thierry, B. Influence of deposition parameters on physico-chemical and optical properties of sputtered PbTiO_3_ thin films. Integr. Ferroelectr., 1996, 13(4), pp. 225–237. https://doi.org/10.1080/10584589608012318, reprinted by permission of Informa UK Limited, trading as Taylor & Francis Ltd., http://www.tandfonline.com on behalf of Jaber, B.; Dogheche, E.; Rèmiens, D.; Thierry, B. Influence of deposition parameters on physico-chemical and optical properties of sputtered PbTiO_3_ thin films. Integr. Ferroelectr., 1996, 13(4), pp. 225–237. https://doi.org/10.1080/10584589608012318.

3. The effect of gas pressure on the (Pb/Ti) ratio of the PT films;

Figure 5, provides the changes in the (Pb/Ti) ratio in the films prepared at *Ts* = 550 °C using a target with x=0.54 at various gas pressures [58]. Under these conditions, stoichiometric thin films are obtained at gas pressures P=50 and 100 mTorr. However, the films prepared at 30 mTorr featured Pb deficiency. Also, films have a highly preferred (111) orientation at 50 mT, at 100 mTorr, a poly structure is observed without any preferred orientations.

4. Dependence of the growth rate on the substrate temperature;

For 25 °C<Ts<720 °C, it has been experimentally verified that while v(*Ts*) remains relatively constant for Ti$O_{2}$ targets, it drastically reduces for the PbO target, when Ts>470 °C. Consistent with (8), variation of the growth rate, with *Ts*, is attributed to re-evaporation of PbO during the film growth in the same manner that (Pb/Ti) depends on *Ts*, as discussed above [71].

**Figure 6:**
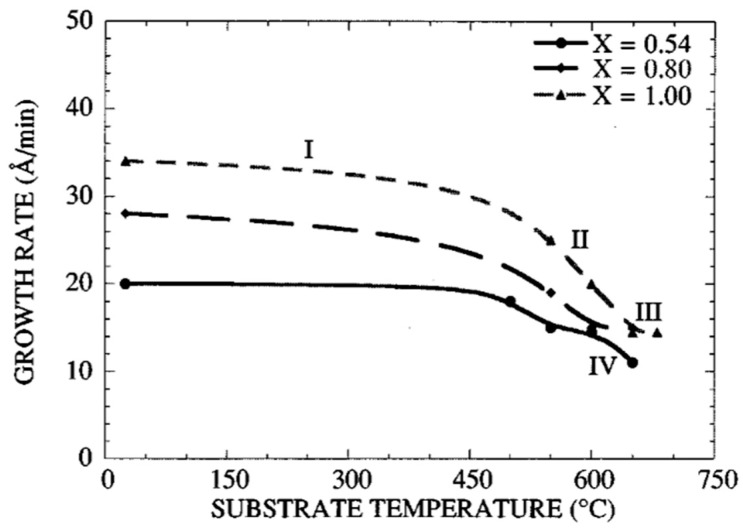
Growth rate of films obtained at various substrate temperatures and target compositions [56]. “Reproduced from Jaber, B.; Remiens, D; Thierry, B. Substrate temperature and target composition effects on PbTiO_3_ thin films produced in situ by sputtering. Journal of Applied Physics, 1996, 79(2), pp. 1182–1184. https://doi.org/10.1063/1.360903, with the permission of AIP Publishing”.

Figure 6 shows the variation of v(*Ts*), for three targets with x=0.54, 0.8, and 1. The variations in v can be divided into four regions: In region I, (0<Ts<500 °C), the value of v is almost independent of *Ts*, while it increases nearly linearly with the increase in x. In region II, v decreases rapidly with *Ts* and the variation is more pronounced for the Pb-rich films (i.e., films obtained from targets with x=0.8 and x=1) [58].

In region III, which exists independent of the value for X, the growth rate approaches a constant value. Note that the degree of PbO re-evaporation from the film depends on the lead content in the target. In Region IV, it is observed only for the target with X =0.54. The variation of the growth rate with the substrate temperature stems from the re-evaporation of lead oxide during the growth. The growth rate decreases above 600 °C. The degree of re-evaporation depends on the lead content in the target and thus the lead content in the film.

5. Dependence of preferential orientation on $\left[{Ar}_{2}/O_{2}\right]$ ratio.

When perovskite films whose polarization-vectors rest along [001] direction are used for pyroelectric applications, it is desired that the films are grown along [001] direction. Since this enhances the film’s pyroelectric coefficient.

Nagao et al. [4] employed magnetron sputtering to prepare perovskite, preferentially c-axis oriented, poly PLT films containing 15% La on Pt/MgO substrate. Their investigations reveal that the orientation of the PLT films is affected by the $\left[{Ar}_{2}\right]/\left[O_{2}\right]$ ratio. Highly (001) oriented PLT films were obtained at $\left[{Ar}_{2}\right]/\left\{\left[{Ar}_{2}\right]+\left[O_{2}\right]\right\}=0.90$. Also, (Pb/Ti) increased linearly and approached a value of 2 at 90% $\left[{Ar}_{2}\right]$. For a 1 micron thick PLT film obtained with $\left[{Ar}_{2}\right]/\left[O_{2}\right]=50/50$, the orientation of the film not only is affected by the compressive stress exerted on the film by the MgO substrate during the cooling period after the termination of the sputtering process but also by the ferroelastic nature of the PLT film. Furthermore, adequate Pb must be supplied to the growing surface to achieve a high-quality c-axis-oriented epitaxial layer, while excessive Pb supply degrades the film quality. They report on achieving films with excellent pyroelectric, dielectric, and ferroelectric behavior.

S. Kim et al. [60] deposited, PT thin films in situ, on MgO single crystalline substrates by the RF magnetron sputtering technique. Films were amorphous for Ts<400 °C, but turned into perovskite structures when Ts>500 °C. Finally, a highly c-axis oriented perovskite structure along the substrate surface normal was obtained with the epitaxial relation PT(100)/MgO(100) when Ts>600 °C. Other deposition parameters were optimized and are presented in Table 2. An increase in the gas pressure from 12 to 24 mTorr, increased the deposition rate but, at the same time, it decreased the degree of c-axis orientation. An increase in the RF power density increased the deposition rate. However, metastable pyrochlore phases were also formed above 3 W/cm^2^, or with pure ${Ar}_{2}$ as the processing gas. The deposition rate decreased significantly with the increase in the $O_{2}$ content of the processing gas. The optimum oxygen content in ${Ar}_{2}$ for stoichiometric PT composition was around 10%. The epitaxial relationship found was PT(100)/MgO(100).

**Figure 7:**
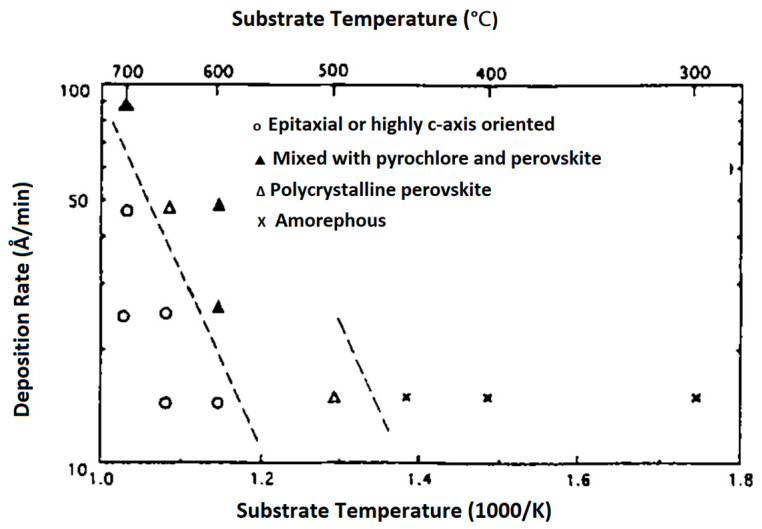
Deposition rate vs. substrate temperature showing the transition from amorphous to poly, and from poly to single crystalline structures for PT thin films (gas composition: $\left[{Ar}_{2}\right]/\left[O_{2}\right]=90/10$, gas pressure: 12 mTorr) [60]. Copyright © Kim, S.; Kang, Y.; Baik, S. Sputter deposition of ferroelectric PbTiO_3_ thin films. Ferroelectrics, 1994, 152(1), pp. 1–6. https://doi.org/10.1080/00150199408017587, reprinted by permission of Informa UK Limited, trading as Taylor & Francis Group, www.tandfonline.com on behalf of Kim, S.; Kang, Y.; Baik, S. Sputter deposition of ferroelectric PbTiO_3_ thin films. Ferroelectrics, 1994, 152(1), pp. 1–6. https://doi.org/10.1080/00150199408017587.

The structural characteristics of PT films as a function of the growth rate and the substrate temperature are summarized in Figure 7, for $\left[{Ar}_{2}\right]/\left[O_{2}\right]=90/10$ at a gas pressure =12 mTorr. Based on the data presented in this figure, for Ts<500 °C, films assume the amorphous phase. For 450 °C<Ts<550 °C, films adopt either a poly perovskite or a perovskite mixed with pyrochlore phase and have 1<v(Ts)<10 nm/min. For 550 °C<Ts<700 °C, films are either single crystalline (epitaxial) or highly c-axis oriented (polycrystalline and) again have growth rates 1<v(Ts)<10 nm/min.

The deposition rates were controlled mainly by varying the RF input power density at Ts>600 °C. Small deposition rates are favorable for the formation of epitaxial or highly c-axis-oriented thin films. From the slope of the broken line in Figure 7, which indicates the transition from polycrystalline to single crystalline structures, the activation energy for epitaxial thin film formation is estimated to be Ea=0.92 eV. 

**Figure 8:**
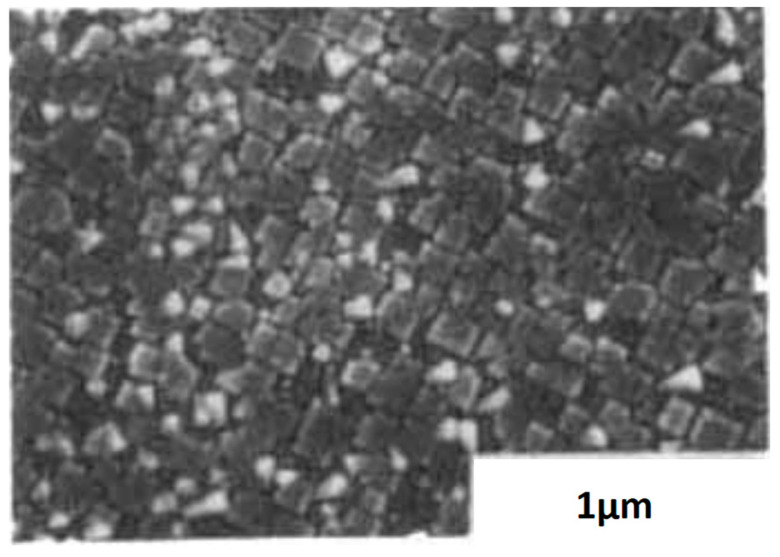
SEM micrograph showing surface morphology of a PT thin film grown in situ at 600 °C on MgO, using a gas composition: $\left[{Ar}_{2}\right]/\left[O_{2}\right]=90/10$ and the gas pressure of 12 mTorr The RF input power density was 2 W/cm^2^ [60]. Copyright © Kim, S.; Kang, Y.; Baik, S. Sputter deposition of ferroelectric PbTiO_3_ thin films. Ferroelectrics, 1994, 152(1), pp. 1–6. https://doi.org/10.1080/00150199408017587, reprinted by permission of Informa UK Limited, trading as Taylor & Francis Group, www.tandfonline.com on behalf of Kim, S.; Kang, Y.; Baik, S. Sputter deposition of ferroelectric PbTiO_3_ thin films. Ferroelectrics, 1994, 152(1), pp. 1–6. https://doi.org/10.1080/00150199408017587.

Figure 8 shows the surface morphology of a PT film grown at 600 °C and input RF power density of 2 Watts/cm^2^. Films adopt a regular mosaic pattern at the surface. Cells are aligned with substrate MgO (100) and typically possess an area of 0.15 μm×0.15 μm.

**Figure 9:**
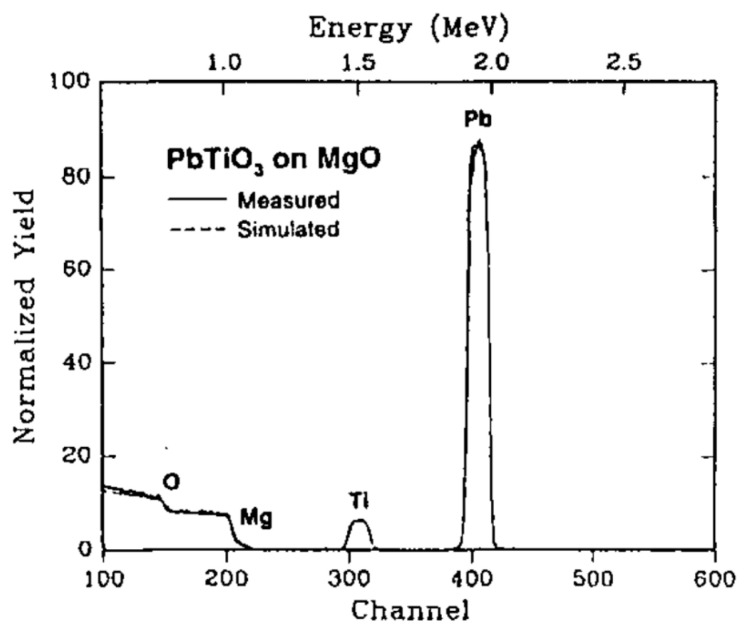
RBS spectrum of a PT thin film on MgO [60]. Copyright © Kim, S.; Kang, Y.; Baik, S. Sputter deposition of ferroelectric PbTiO_3_ thin films. Ferroelectrics, 1994, 152(1), pp. 1–6. https://doi.org/10.1080/00150199408017587, reprinted by permission of Informa UK Limited, trading as Taylor & Francis Group, www.tandfonline.com on behalf of Kim, S.; Kang, Y.; Baik, S. Sputter deposition of ferroelectric PbTiO_3_ thin films. Ferroelectrics, 1994, 152(1), pp. 1–6. https://doi.org/10.1080/00150199408017587.

Rutherford backscattering (RBS) of Figure 9 confirms a stoichiometry close to that of the target material.

#### 4.3.2. Solid State Crystallization

A second route to preparing members of the PT family perovskites is the low-temperature deposition (*Ts* < Tcrys) of the amorphous PT by magnetron sputtering, at first, and then heat treatment in $O_{2}$(post-annealing). Post-annealing is needed because the phase transition in PT ceramics requires high spontaneous lattice strain. This renders the room-temperature fabrication of such ceramics impractical. Both poly and amorphous phases convert into the single crystalline phase at *Ts* > Tcrys, by heat treatment, or laser annealing in $O_{2}$. The processing temperature in such cases is the crystallization temperature, Tcrys. This technique, referred to as solid-state crystallization (epitaxy), leads to the improvement of the dielectric properties of such films [14,50,60,61,62]. Magnetron sputtering conditions commonly utilized in the preparation of PT family films prepared by solid-state crystallization are summarized in Table 2.

In Table 2(b), it provides processing parameters utilized in the preparation of PT family films by solid-state crystallization of the same films outlined in Table 2(a).

By suitable choice of substrate and the processing conditions, it is generally possible to transform a film material from an amorphous phase to poly or from poly to a single crystalline structure.

Matsui et al. [72] used RF sputtering to deposit PT films at low temperatures (350~450 °C). These films were then annealed in the furnace at 400–900 °C or under irradiation of CW ${CO}_{2}$ laser at 10–40 W for 1–10 s. in Table 3. It was found from the X-ray diffraction analysis that the film quality improved over time and by increasing temperature.

In ref. [73], the target material was a mixed, PbTi$O_{3}$ and PbZr$O_{3}$ and material powder with 10 w% excess PbO. The target composition was chosen near the morphotropic phase boundary.

S. Kim et al. [59] deposited thin films of amorphous PT, on Pt substrates using deposition parameters outlined in Table 2(a). The isothermal treatments for crystallization were conducted in air in a tube furnace. Polycrystalline PT, thin films were obtained by crystallizing amorphous films according to the conditions outlined in Table 2(b). The isothermal crystallization kinetic study carried out at 475 °C predicts growth mechanism as discussed below:

Various phase transformation behaviors are identified by their Avrami constant (n). The volume fraction of a specific crystalized phase (x), in the grown film is determined by integrating X-ray intensities of the main reflections (e.g., for the perovskite PT Phase, the main reflections are the (101) and (110)). The isothermal phase transformation is described by Johnson–Mehl–Avrami via [43]:

(10) $$x=1-\exp\left[-{\left(kt\right)}^{n}\right],$$

where x is the volume fraction of the crystalized phase, k is the rate constant $\left(s^{-1}\right)$, t is the time (s). The Averami constant n is readily found from the slope of $\ln\left(-\ln\left(1-x\right)\right)$ versus $\ln\left(t\right)$, and is indicative of the behavior of the phase transformation as described in Table 3 [76].

**Figure 10:**
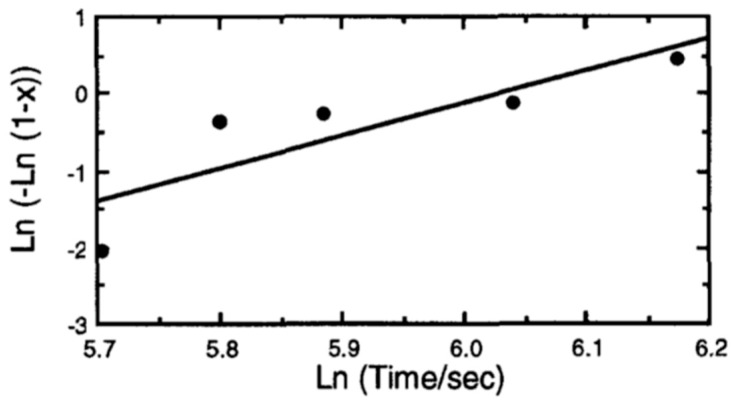
Johnson–Mehl–Avrami plot of crystallization of isothermal treated PT, thin films on Pt substrates at 475 °C [60]. Copyright © Kim, S.; Kang, Y.; Baik, S. Sputter deposition of ferroelectric PbTiO_3_ thin films. Ferroelectrics, 1994, 152(1), pp. 1–6. https://doi.org/10.1080/00150199408017587, reprinted by permission of Informa UK Limited, trading as Taylor & Francis Group, www.tandfonline.com on behalf of Kim, S.; Kang, Y.; Baik, S. Sputter deposition of ferroelectric PbTiO_3_ thin films. Ferroelectrics, 1994, 152(1), pp. 1–6. https://doi.org/10.1080/00150199408017587.

Figure 10 shows the result of the Johnson–Mehl–Avrami plot. The slope found by the least-square fit method, gives the Avarami constant, *n*~4, which implies that the general mechanism governing crystallization from amorphous to perovskite phase is interface reaction controlled, characterized by the “isotropic 3-dimensional growth of nuclei” and is formed with constant nucleation rate.

**Figure 11:**
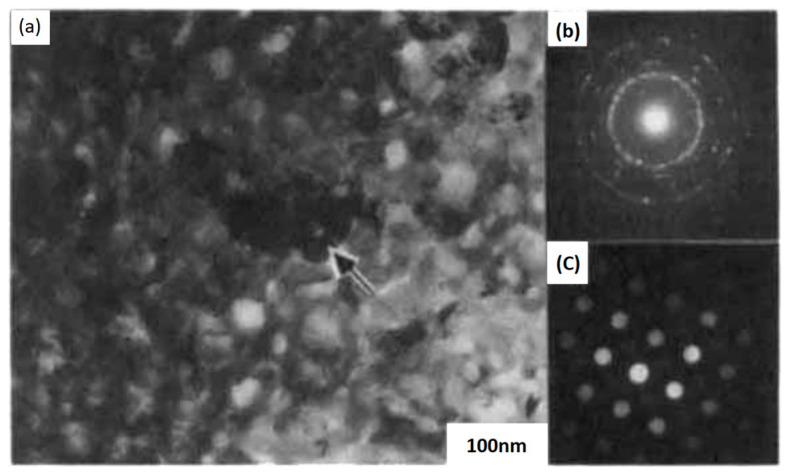
TEM micrographs of fully crystallized (550 °C, 5 min) PT Thin film on Pt: part (**a**) bright field image, part (**b**) selected area diffraction pattern, and part (**c**) micro-diffraction pattern of the arrowed grain in (**a**) [60]. Copyright © Kim, S.; Kang, Y.; Baik, S. Sputter deposition of ferroelectric PbTiO_3_ thin films. Ferroelectrics, 1994, 152(1), pp. 1–6. https://doi.org/10.1080/00150199408017587, reprinted by permission of Informa UK Limited, trading as Taylor & Francis Group, www.tandfonline.com on behalf of Kim, S.; Kang, Y.; Baik, S. Sputter deposition of ferroelectric PbTiO_3_ thin films. Ferroelectrics, 1994, 152(1), pp. 1–6. https://doi.org/10.1080/00150199408017587.

Figure 11 shows the microstructure of a fully crystallized PbTi$O_{3}$ thin film. The bright field image taken with the plane view TEM in Figure 11 shows that the crystallized films are composed of very fine grains on average 20–100 nm in size.

Figure 11 is a selected area diffraction pattern revealing a ring pattern, which indicates that the grains are randomly oriented. There exists no evidence of 90°-domain boundaries within individual grains.

The effect of substrate and processing conditions on the film morphology is further discussed in Section 4.

In order to control the stoichiometric composition of PLZT film during the growth, Wasa et al. [62] utilized a three-target (made of Pb, Ti, or La) sputtering system, where each magnetron cathode was powered by a separate DC power supply. The process was carried out in an oxidizing ambient environment, and the film composition could be controlled by controlling the sputtering rate of each target. 

**Figure 12:**
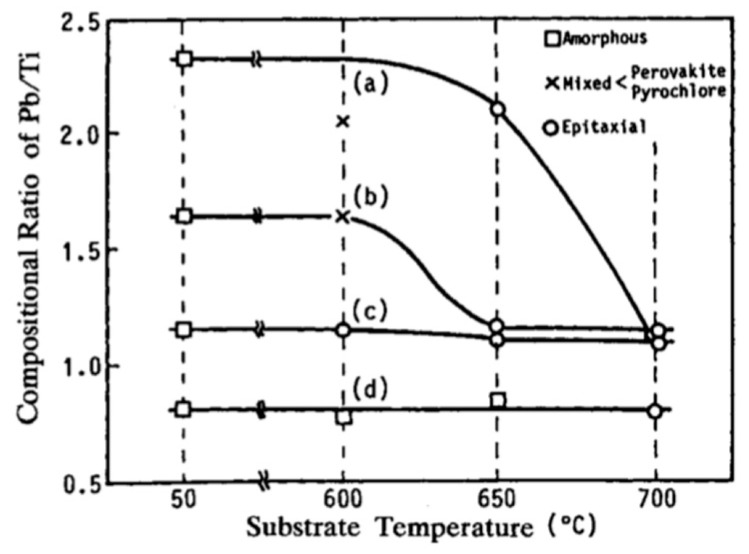
Composition and crystallinity of the PLZT films are obtained at various substrate temperatures and incident (Pb/Ti) ratios. The Pb/Ti ratios: (a) 2.3, (b) 1.6, (c) 1.2, (d) 0.8 from Wassa et al. [62]. “Copyright © Wasa, K.; Adachi, H.; Kitabatake, M. Sputtering deposition process of perovskite Pb-Ti-O_3_ families. Ferroelectrics, 1992, 137(1), pp. 343–356. https://doi.org/10.1080/00150199208015965, reprinted by permission of Informa UK Limited, trading as Taylor & Francis Ltd., http://www.tandfonline.com on behalf of Wasa, K.; Adachi, H.; Kitabatake, M. Sputtering deposition process of perovskite Pb-Ti-O_3_ families. Ferroelectrics, 1992, 137(1), pp. 343–356. https://doi.org/10.1080/00150199208015965”.

Wasa et al. [62] studied the composition and crystallinity of PLZT films obtained at various substrate temperatures and incident (Pb/Ti) ratios. Figure 12 shows the variation of the (Pb/Ti) and crystalline quality of the sputtered PLZT films as a function of *Ts*. Since re-evaporation is negligible at low temperatures, the (Pb/Ti) ratio for the films grown at 50 °C is regarded as the ratio of the “actual” (Pb/Ti), incident to the substrate. The decline in the (Pb/Ti) ratio at higher temperatures is a consequence of the re-evaporation during the film growth. The degree of the re-evaporation depends on the (Pb/Ti) ratio. Re-evaporation of an element or a molecule from the film decreases the substrate temperature at which the film grows with the perovskite structure and induces a specific orientation in the growing film.

From Figure 12, the vapor phase epitaxial temperature, i.e., substrate temperature for the epitaxial growth, Tepi, is around 600 °C. When 1.1<(Pb/Ti)<1.2, a wide temperature window for the epitaxial growth exists (600 °C<Tepi<700 °C). The excess Pb in the sputtered films is transported to and localized around the grain boundaries of the perovskite crystallites. Any increase in the oxidation rate of the Pb adatoms during film growth reduces the re-evaporation rate of the Pb adatoms. According to Schlom et al. [67] the amount of the Pb re-evaporation diminishes when the gas mixture includes highly oxidizing gas(es) such as ozone and/or N_2_O. 

**Figure 13:**
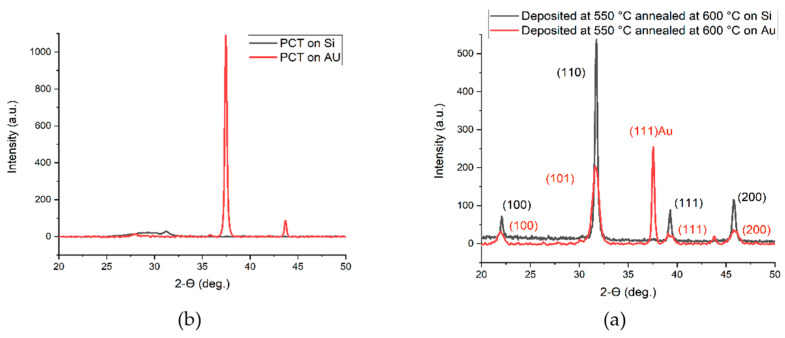
(**a**,**b**) show the XRD data of PCT thin films deposited on Si and Si/SiN/Ti/Au substrates before and after annealing, respectively [16]. “Mafi, E.; Calvano, N.; Patel, J.; Islam, M. S.; Hasan Khan, M. S.; Rana, M. (2020). Electro-Optical Properties of Sputtered Calcium Lead Titanate Thin Films for Pyroelectric Detection. *Micromachines*, **2020**, *11(12)*, 1073. https://doi.org/10.3390/mi11121073.”, retrieved from https://doi.10.3390/mi11121073, used under Creative Commons Attribution-4.0 International(https://creativecommons.org/licenses/by/4.0/).

**Figure 14:**
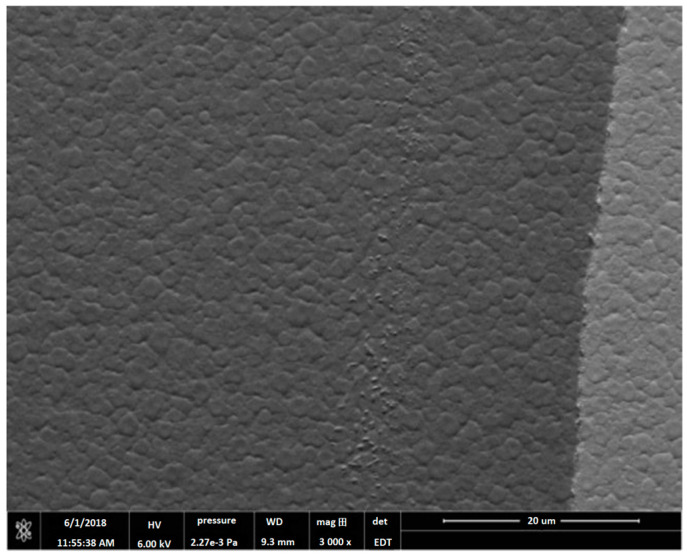
An SEM micrograph showing crystal growth PCT thin film annealed at 600 °C [16]. “Mafi, E.; Calvano, N.; Patel, J.; Islam, M. S.; Hasan Khan, M. S.; Rana, M. (2020). Electro-Optical Properties of Sputtered Calcium Lead Titanate Thin Films for Pyroelectric Detection. *Micromachines*, **2020**, *11(12)*, 1073. https://doi.org/10.3390/mi11121073.”, retrieved from https://doi.10.3390/mi11121073, used under Creative Commons Attribution-4.0 International (https://creativecommons.org/licenses/by/4.0/).

E. Mafi et al. [16] reported on the preparation and characterization of crack-free PCT thin films with smooth surfaces for use in pyroelectric detectors. PCT films were deposited on both silicon and Si/SiN/Ti/Au substrates at 13 mTorr pressure of a gas-containing mixture of ${Ar}_{2}$ and $O_{2}$ using a 200 W-RF sputtering source for four hours at 550 °C<Ts<800 °C. The PCT films were then annealed at Tpan=550, 600, 650 and 700 °C in $O_{2}$ environment for small time intervals of 15 min. Their XRD analysis confirmed the polycrystalline nature of these films. Energy dispersive spectroscopy (EDS) revealed that the films were stoichiometric ${Ca}_{0.43}{Pb}_{0.57}$Ti$O_{3}$. The film thicknesses varied from ~ 250 to 400 nm. They find that films deposited at 550 °C and 600 °C demonstrate higher quality dielectrics with a larger value of the pyroelectric coefficient. The capacitors fabricated using the PCT films at 550 °C showed pyroelectric current as high as 14 pA and pyroelectric coefficients as high as 50 μC/m^2^K. Figure 13 shows the XRD of the thin films while Figure 14 shows the SEM micrograph of surface topography.

K. Sreenivass et al. [50] investigated the structural, electrical, and optical properties of reactive D.C. -magnetron-sputtered Pb${Zr}_{x}{Ti}_{1-x}O_{3}$ or (PZT) thin films prepared using a multielement metallic target and studied the influence of the growth conditions on the surface morphology of such films. As-grown deposits consist of a complex mixture of the suboxides of lead, zirconium, and titanium. The substrate temperature and the oxygen partial pressure are the main control parameters of this process. Single-phase PZT forms by post-oxidation a diffusion-controlled process on the substrate. The crystallographic and electrical properties are determined by the (Zr/Ti), as well as the relative oxidation and diffusion rates, which result in the incorporation of PbO into a complex matrix of Zr$O_{2}$ and Ti$O_{2}$. 

Pignolet et al. [14] studied the structure and characteristics of the RF-magnetron sputtered pure and doped PT as well as the PZT thin films deposited on platinum-coated silicon by RF-magnetron sputtering from a pressed powder target. Depending on the composition, poly films with either a tetragonal or rhombohedral structure were obtained using only a post-deposition thermal treatment (in the absence of any in situ heat treatment) see Table 2. They find that the temperature required to obtain the ferroelectric phase depends on the chemical composition of the film. For pure PT films, the annealing temperature is 800 °C<Tpan<820 °C, and yields a tetragonal structure. The films exhibited ferroelectric structure after only 5 to 10 min of annealing. Yet, the electrical properties improve by a longer anneal period of 3 to 5 h.

For the pure PT, the post-annealing temperature is 800 °C<Tpan<820 °C, and for PZT films, it is 600 °C<Tpan<650 °C. For pure PT and PZT, with less than 40 mol.% zirconium, the structure is tetragonal, while for PZT with more than 50 mol.% Zr, it is rhombohedral. In between lies the morphotropic phase boundary (MPB), confirming that the composition determines the crystalline structure of the ferroelectric phase. Similar to observations made on films prepared using in situ heat treatment, the presence of PbO in the target powder has little influence on the film structure except that films sputtered from PbO-enriched targets show smaller concentrations of undesirable phases such as pyrochlore.

## 5. Discussion and Results

Magnetron sputtering is essentially a non-equilibrium process. Various phases evolve under different sets of processing parameters. Heteroepitaxial thin films of perovskite materials consist of microstructures that are governed by the initial stage of the film growth. The deposited PT thin films with thicknesses 0.5<t<1.5 μm are white or yellowish and transparent. The films with the pyrochlore ${Pb}_{2}{Ti}_{2}O_{6}$ phase have an intense yellow color. PT has a cubic perovskite-type structure above the Tc (=490 °C). Below Tc, it transforms into a tetragonal lattice. 

The room-temperature lattice parameters of the tetragonal structure are a=0.3904 nm and c=0.4152 nm [47,61,77,78]. This results in an anisotropy that develops during cooling through the cubic-tetragonal phase and is measured by the tetragonality of the unit cell c/a (~1.06). A large anisotropy reflects enhanced pyroelectric behavior [79]. 

Jangade et al. [80] prepared bulk poly PT with grain sizes ranging from 1 to 2 μm by solid-state reaction. 

A number of practical parameters are of prime importance in defining the morphology of the magnetron-sputtered PT thin films. These include substrate/buffer layer crystalline quality, growth temperature, deposition rate, processing gas composition and pressure, R.F. power, applied negative bias to the substrate, cooling rate, and extra PbO content in the source. It is constructive to estimate what might be expected from a set of process variables and to understand how such parameters may be manipulated to achieve a desired phase. Some of the practical factors that affect the characteristics of the thin film epitaxial growth are discussed below.

### 5.1. The Crystalline Quality of the Substrate/Buffer Layer

**Figure 15:**
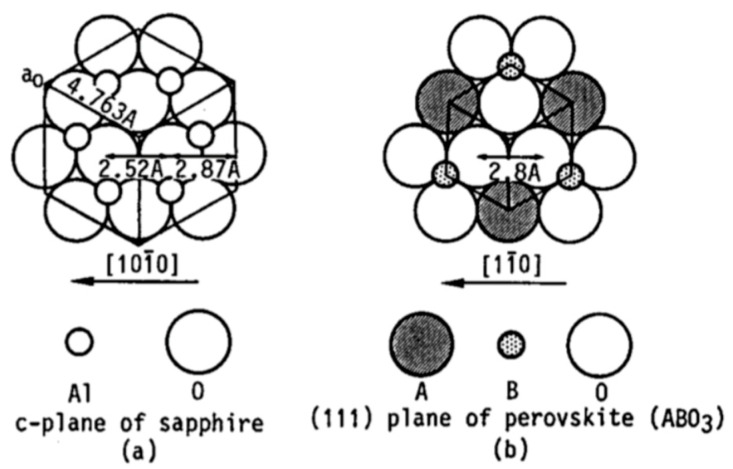
Part (**a**) and part (**b**) are Planar atomic arrangement of the c-plane of sapphire and the (111) plane of cubic perovskite (AB$O_{3}$) [62]. “Copyright © Wasa, K.; Adachi, H.; Kitabatake, M. Sputtering deposition process of perovskite Pb-Ti-O_3_ families. Ferroelectrics, 1992, 137(1), pp. 343–356. https://doi.org/10.1080/00150199208015965, reprinted by permission of Informa UK Limited, trading as Taylor & Francis Ltd., http://www.tandfonline.com on behalf of Wasa, K.; Adachi, H.; Kitabatake, M. Sputtering deposition process of perovskite Pb-Ti-O_3_ families. Ferroelectrics, 1992, 137(1), pp. 343–356. https://doi.org/10.1080/00150199208015965”.

To realize high-quality epitaxial films, it is crucial to start with a single crystalline substrate or a substrate covered with a buffer (intermediate) layer having proper symmetry, orientation, lattice constant, chemical nature, and thermal coefficient of expansion (TCE). Lattice mismatch between the film and substrate must be kept small (less than 10%). Sapphire (${Al}_{2}O_{3}$), magnesium oxide (MgO), strontium oxide (SrTi$O_{3}$), and Platinum (Pt) have frequently been used as substrates for the epitaxial growth of PT films. Typical atomic planar arrangements of the cubic perovskite (AB$O_{3}$) and the c-plane of sapphire are shown in Figure 15 [62].

Table 4 gives relevant crystallographic information along with TCE for the PT, as well as several other important substrates.

**Figure 16:**
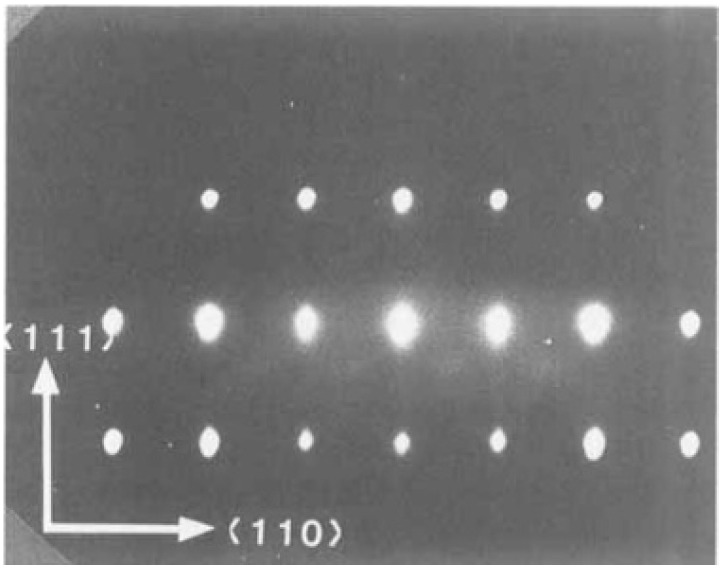
Typical REED patterns of PLZT (28/0/100) thin films of 0.4 μm thick deposited on (0001) sapphire, Wassa et al. [62]. “Copyright © Wasa, K.; Adachi, H.; Kitabatake, M. Sputtering deposition process of perovskite Pb-Ti-O_3_ families. Ferroelectrics, 1992, 137(1), pp. 343–356. https://doi.org/10.1080/00150199208015965, reprinted by permission of Informa UK Limited, trading as Taylor & Francis Ltd., http://www.tandfonline.com on behalf of Wasa, K.; Adachi, H.; Kitabatake, M. Sputtering deposition process of perovskite Pb-Ti-O_3_ families. Ferroelectrics, 1992, 137(1), pp. 343–356. https://doi.org/10.1080/00150199208015965”.

**Figure 17:**
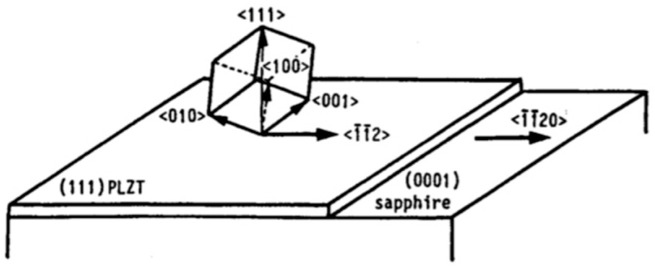
Crystal orientation of the epitaxial (111) PLZT film on (0001) sapphire Wassa et al. [62]. “Copyright © Wasa, K.; Adachi, H.; Kitabatake, M. Sputtering deposition process of perovskite Pb-Ti-O_3_ families. Ferroelectrics, 1992, 137(1), pp. 343–356. https://doi.org/10.1080/00150199208015965, reprinted by permission of Informa UK Limited, trading as Taylor & Francis Ltd., http://www.tandfonline.com on behalf of Wasa, K.; Adachi, H.; Kitabatake, M. Sputtering deposition process of perovskite Pb-Ti-O_3_ families. Ferroelectrics, 1992, 137(1), pp. 343–356. https://doi.org/10.1080/00150199208015965”.

Wasa et al. [62] epitaxially grew members of PbTi$O_{3}$, family including (111) oriented PT, (Pb,Zr)Ti$O_{3}$, and (Pb,La)(Zr,Ti)$O_{3}$ on (0001) sapphire. A typical Reflection High-Energy Electron Diffraction (RHEED) pattern and crystal orientation are shown in Figure 16 and Figure 17, respectively. Also, Wasa et al. [62] grew epitaxial (100) PT thin films on the (100) MgO and/or (100) SrTi$O_{3}$ single crystal substrates.

Wang et al. [73] prepared PZT in the composition range near the morphotropic transition boundary (x=0.53) thin films by R.F. magnetron sputtering on (100) silicon and sapphire. Table 5 gives the lattice constant and the O-O spacing for the sputtered films of the PbTi$O_{3}$ families. The average O-O spacing for the (0001) sapphire is 2.75 A°, hence, the lattice mismatch is around 5%. Consequently, excellent epitaxial growth on the sapphire is possible. 

The optimized conditions for the sputtering and post-deposition annealing are given in Table 3, respectively. The as-grown films are amorphous or crystalline with the pyrochlore structure. Crystalline phase transformation from an amorphous or a pyrochlore to a perovskite-structures occurs around 600 °C for all the PZT (x=0.53) thin films. The necessary post-deposition annealing time increases if the deposition temperature increases. 

The stress induced in the films due to the lattice mismatch is localized in the interface region between the substrate surface and the deposited films. For thin films 0.1–2 μm thick, the interface region thickness is on the order of nanometers and, therefore, is not expected to modify the dielectric properties of the film.

Okuyama et al. [47] prepared epitaxial PT films on MgO substrate. Lattice mismatches of MgO with a- and c-axis PT are only 7.9% and 1.5 %, respectively. However, it suffers from its hydroscopic nature. 

**Figure 18:**
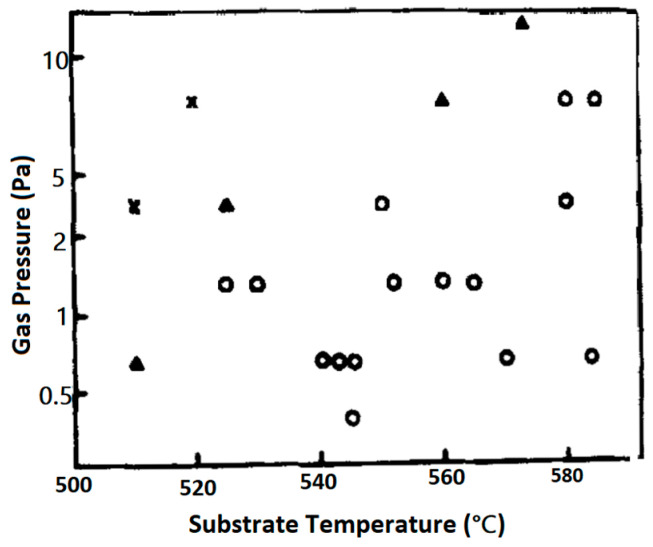
Pressure-temperature phase diagram for PbTi$O_{3}$ on corning 7059 glass substrate (°, perovskite; x, pyrochlore and Δ shows mixed perovskite-pyrochlore phase) [46]. “Copyright © Shiosaki, T.; Adachi, M.; Mochizuki, S.; Kawabata, A. Properties of sputter-deposited PbTiO_3_, Pb (Zr, Ti) O_3_, Pb_2_KNb_5_O_15_ films. Ferroelectrics, 1985, 63(1), pp. 227–234. https://doi.org/10.1080/00150198508221404, reprinted by permission of Informa UK Limited, trading as Taylor & Francis Group, www.tandfonline.com on behalf of Shiosaki, T.; Adachi, M.; Mochizuki, S.; Kawabata, A. Properties of sputter-deposited PbTiO_3_, Pb (Zr, Ti) O_3_, Pb_2_KNb_5_O_15_ films. Ferroelectrics, 1985, 63(1), pp. 227–234. https://doi.org/10.1080/00150198508221404”.

Wasa et al. [62] studied the effects of processing variables on the morphology of the PT films, grown on MgO substrates. Thin PT films grown below 400 °C adopt the amorphous phase, while those grown above 500 °C can obtain the perovskite phase. At 600 °C a highly c-axis oriented epitaxial film is obtained with epitaxial relation (100)PT/(100) MgO. Shiosaki et al. [46] prepared polycrystalline and epitaxial films of PT and Pb(Zr,Ti)$O_{3}$ in perovskite phase on the glass, platinum, and sapphire substrates by RF planar magnetron sputtering, using ceramic targets. The map shown in Figure 18 depicts the conditions in the P-Ts domain, under which PT films grow in the perovskite phase on the Corning 7059 glass substrates. It is seen that for the in situ growth (without post-thermal annealing), the perovskite phase is obtained when Ts>530 °C.

Also, using nearly the same conditions as those used in Figure 18, they grew polycrystalline perovskite films, as well as the epitaxial films of pure (101), mixed (101)-(110), and pure (111) orientations in the perovskite phase on the R (01$\bar{1}$2) face of sapphire ($\alpha -{Al}_{2}O_{3}$) at 540 °C<Ts<560 °C. 

For the PZT films sputter-deposited at 400 °C<Ts<550 °C, Shiosaki et al. [46] reported pure pyrochlore or perovskite-pyrochlore mixed phase, which then changed to the perovskite phase when annealed at 600 °C<Ts<800 °C for 2 to 6 h. However, the pyrochlore films deposited at Ts>550 °C hardly changed the phase by post-thermal annealing.

Ogawa et al. [84] deposited thin PT film by RF magnetron sputtering on various substrates to investigate the effect of substrate thermal expansion coefficient on the orientation of the film. They find that if the TCE of the substrate is smaller than the TCE of the film, as is the case of a quartz glass substrate with PT film, tensile stress in the film results in an a-axis orientation of the film. On the other hand, if the thermal expansion coefficient of the substrate is larger than that of the film, as is the case of a single-crystal MgO substrate with PT film, the horizontal compressive mechanical stress along the plane of the film gives rise to a c-axis-orientation of the film. The Curie point of the film material determines the amount of mechanical stress present during the cooling. 

Kushida et al. [85] used a seeded lateral overgrowth technique to prepare c-axis oriented PT films, on a Pt electrode patterned (l00) single-crystal ST substrate. ST is chosen because it has the same crystal structure as PT and its lattice constant matches well with that of the a-axis PT (see Table 5). Sputtering conditions are summarized in Table 2. As expected, PT film epitaxially grew on the ST single crystal and extended laterally over the Pt electrode. The portion of the PT film which lay only on the Pt electrode film was found to be polycrystalline with grains as large as 0.2 μm. However, when the film grew to 1.5 μm thick, the polycrystalline portion completely converted to an epitaxial film.

Okuyama et al. [47,49] deposited PT films on a Pt sheet at a substrate temperature range of 440 °C–450 °C, and Iijima et al. [57] used PT powder with 20 mol.% excess PbO to deposit (100) and (001) PT films on MgO at Ts=575 °C under P=2 Pa to grow epitaxial PbTi$O_{2}$ thin films on MgO single crystal and epitaxial Pt film substrates by the RF-magnetron sputtering at a deposition rate <2 nm/min and low gas pressure (∼1 Pa) using a PbO-rich target (Table 2). Films were found to feature 98% c-axis orientation. Furthermore, the c-axis of the tetragonal phase was parallel to the substrate surface just below Tc and the c-axis becomes perpendicular to the substrate surface as the temperature is lowered. This technique yielded a small deposition rate. Moreover, PT films deposited on an oriented Pt inter-layer grown on MgO crystal were also oriented at (001) and (100).

Buffer layers are commonly used between the substrate and the growing film to minimize various adverse effects such as unwanted interfacial oxide growth and substrate-film lattice mismatch, as well as to reduce inter-diffusion of elements during epitaxy and to enhance film crystallization. The c-axis-oriented poly or epitaxial PT films have shown fascinating ferroelectric properties. Yet, in lieu of the need for their integration with the integrated circuit (IC) processing technology, it is desired that these films be deposited on Si substrates. However, during the first stages of the sputtering of such films, an amorphous thin interfacial silicon oxide grows on Si which prohibits subsequent growth of crystalline PT. A large mismatch in the TCE of the substrate and the film affects the mechanical integrity of the films. Thin buffer layers are effective in mitigating the adverse effects of TCE mismatch between the film and the substrate and can promote adhesion and support smooth film surfaces. The surface morphology of the films depends on processing conditions and the type of substrate used. Hillock formation is primarily attributed to stress relief during thermal cycling. K. Sreenivass et al. [50] note that the use of low substrate temperatures (200 °C) during sputter deposition of the film and long annealing periods (20 h) leads to smooth surface morphology and prevents microcracking in the films. 

When the lattice mismatch between the growing film and the substrate is large, a buffer layer with proper lattice constant can be utilized to enhance the film’s crystalline quality. As an example, consider the question regarding the viability of using a Pt interlayer as a buffer layer for the growth of epitaxial PZT film on (0001) sapphire [64]. The PZT (90/10), i.e., ${PbZr}_{x}{Ti}_{1-x}O_{3}$ belongs to a rhombohedral structure and has a polar axis parallel to the [111]. The lattice mismatch between the PZT film and the sapphire substrate is 6.2%, which is much larger than that of PT film and the same substrate (1.8%), in the triangular arrangement of oxides perpendicular to their three-fold axes. Furthermore, the lattice constant of PZT increases with increasing Zr concentrations. On the other hand, Pt has the same structure as PZT with a point group m3m at 600 °C. The surface normal axis of the PZT, (111) Pt, and (0001) sapphire possess 3-fold properties and the lattice mismatch between (111) Pt, and (0001) sapphire is 1.1%, This information is summarized in Table 6, suggesting that Pt is in fact a viable choice for this case since the mismatch between (111) PZT and (111) Pt is 5%, a value smaller than that of PZT and (0001) sapphire.

**Figure 19:**
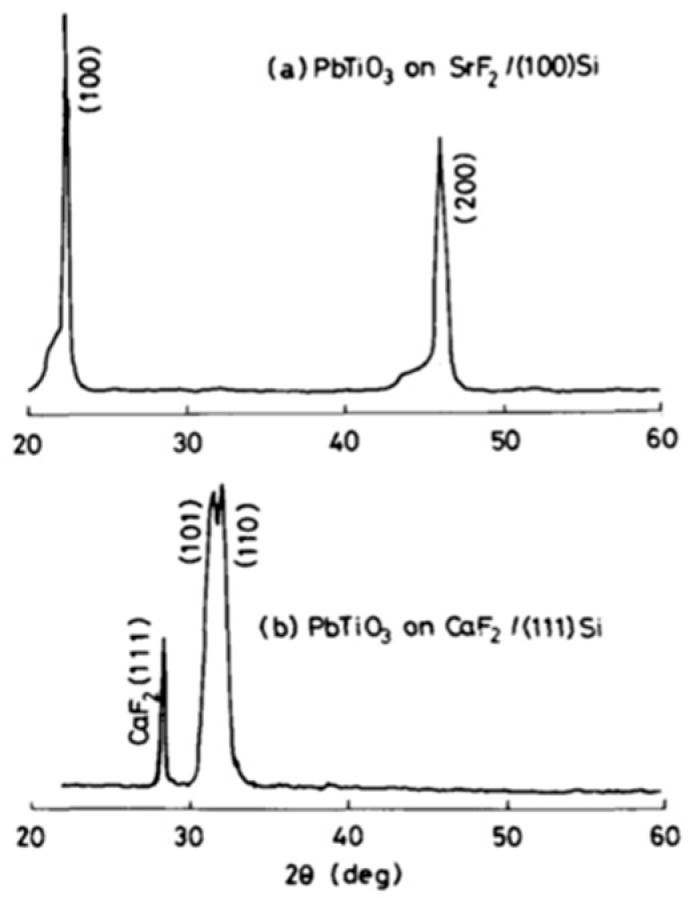
(Cu-Kα) X-ray diffraction pattern of the PT thin films deposited on ${\mathrm{Ca}F}_{2}$ and ${\mathrm{Sr}F}_{2}$ films on Si [55]. “Copyright © Okuyama, M.; Ueda, T.; Hamakawa, Y. Preparation of Oriented PbTiO_3_ Ferroelectric Thin Films on Silicon. The Japan Society of Applied Physics, vol. 24, no. S2, p. 619, Jan. 1985, https://doi.org/10.7567/JJAPS.24S2.619. Page: 620 Figure 1. The Japan Society of Applied Physics. Reproduced by permission of IOP Publishing Ltd. All rights reserved”.

Lattice mismatches of (111) Si with ${CaF}_{2}$ and ${SrF}_{2}$ films have been reported as 6% and 6.8%, respectively. Furthermore, they both are crystalline films remarkably tolerant to oxidation and thus can be regarded as suitable buffer layers between Si and PT film. Okuyama et al. [55] prepared oriented PT ferroelectric thin films prepared on (111)/(100) Si wafer by RF sputtering. Highly-oriented ${CaF}_{2}$ and $SrF_{2}$ films with excellent crystalline properties on Si wafer could be grown to serve as buffer layers. These films were prepared by electron beam evaporation at 400 °C–600 °C. The PT film on the ${SrF}_{2}$/(100) Si had (100) orientation, but the ones grown on ${CaF}_{2}$ were oriented in (111) (110) and (101) directions. These claims are supported by the data in Figure 19, which shows the X-ray diffraction pattern of PT films deposited on ${SrF}_{2}$/(100) Si and ${CaF}_{2}$/(111) Si. The PT film on the ${CaF}_{2}$/(111) Si is oriented to (110) and (101).

**Figure 20:**
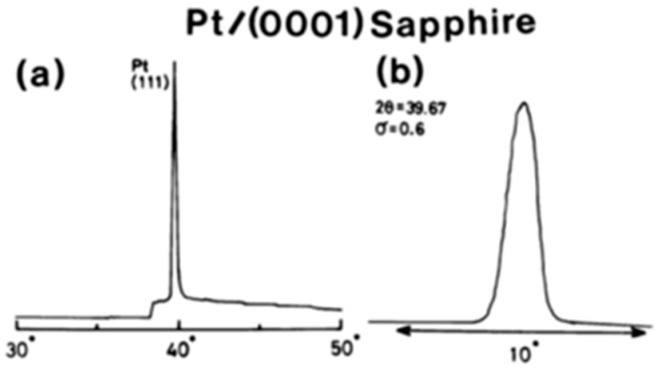
Part (**a**) and (**b**) is X-ray diffraction pattern of the epitaxial Pt thin film on the (0001) sapphire [64]. “Copyright © Adachi, M.; Matsuzaki, T.; Yamada, T.; Shiosaki, T.; Kawabata, A. Sputter-Deposition of [111]-Axis Oriented Rhombohedral PZT Films and Their Dielectric, Ferroelectric and Pyroelectric Properties. Jpn. J. Appl. Phys., 1987, 26 (Part 1, No. 4), pp. 550–553. https://doi.org/10.1143/JJAP.26.550. Page: 551 Figure 1. Japanese Journal of Applied Physics: JJAP online. Reproduced by permission of IOP Publishing Ltd. All rights reserved.”.

**Figure 21:**
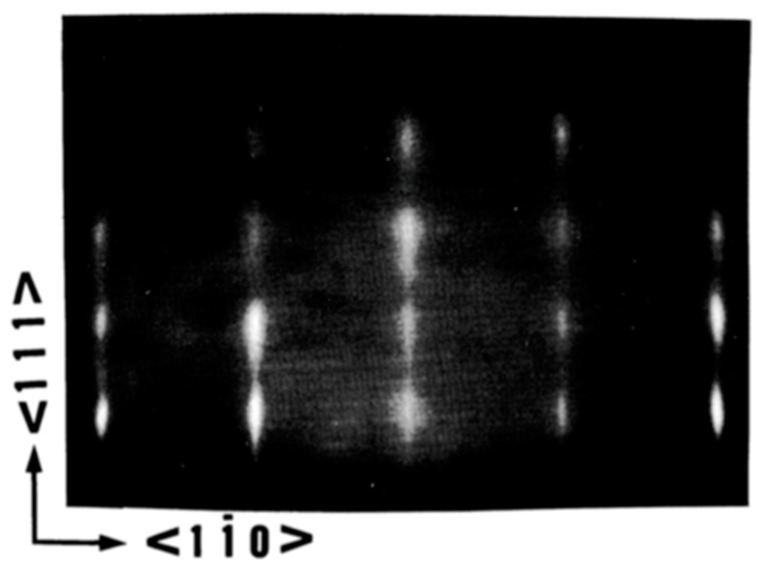
RHEED pattern of an epitaxial Pt thin film on (0001) sapphire [64]. “Copyright © Adachi, M.; Matsuzaki, T.; Yamada, T.; Shiosaki, T.; Kawabata, A. Sputter-Deposition of [111]-Axis Oriented Rhombohedral PZT Films and Their Dielectric, Ferroelectric and Pyroelectric Properties. Jpn. J. Appl. Phys., 1987, 26 (Part 1, No. 4), pp. 550–553. https://doi.org/10.1143/JJAP.26.550. Page: 551 Figure 2. Japanese Journal of Applied Physics: JJAP online. Reproduced by permission of IOP Publishing Ltd. All rights reserved”.

**Figure 22:**
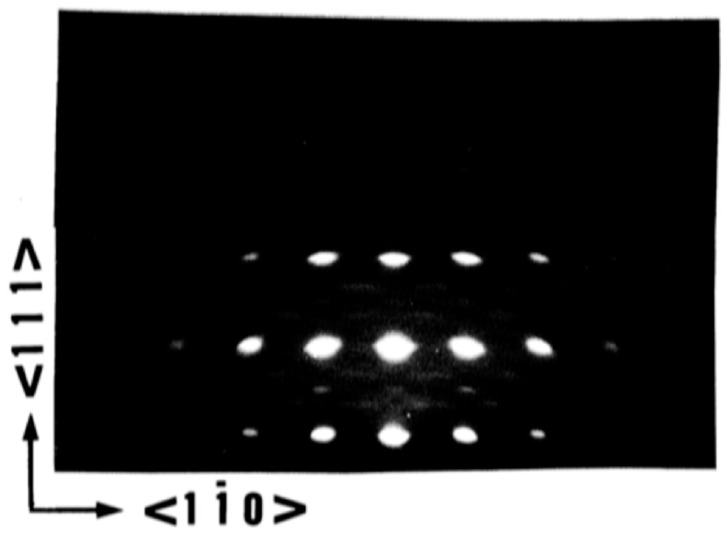
RHEED Pattern of an epitaxial PZT film on Pt/Sapphire [64]. “Copyright © Adachi, M.; Matsuzaki, T.; Yamada, T.; Shiosaki, T.; Kawabata, A. Sputter-Deposition of [111]-Axis Oriented Rhombohedral PZT Films and Their Dielectric, Ferroelectric and Pyroelectric Properties. Jpn. J. Appl. Phys., 1987, 26 (Part 1, No. 4), pp. 550–553. https://doi.org/10.1143/JJAP.26.550. Page: 551 Figure 4. Japanese Journal of Applied Physics: JJAP online. Reproduced by permission of IOP Publishing Ltd. All rights reserved”.

These crystalline orientations can be explained in terms of prevalent lattice mismatches. M. Adachi et al. [64] sputter deposited [111]-axis-oriented rhombohedral PZT films onto epitaxial (111) platinum/(0001) sapphire substrate, by RF-magnetron sputtering using PbO enriched PZT (90/10) targets according to the recipe described Table 2a,b. At Ts<580 °C, a metastable pyrochore phase appeared. PZT of perovskite structure was obtained at Ts=580 °C. The X-ray diffraction pattern and the rocking curve of an epitaxial (111) PZT film sputtered on the (111) Pt/(0001) sapphire grown in situ at Ts=610 °C are shown here. The rocking curve of Figure 20, which depicts high quality (111) diffraction patterns, with standard deviation angle, σ=0.6°, together with the high energy electron diffraction (RHEED) patterns of Figure 21 and Figure 22, which display sharp diffraction spots, substantiate the possibility of growing high quality epitaxial (111) Pt on (0001) sapphire and using this structure for the epitaxial growth of PZT film. Furthermore, the following epitaxial relations were confirmed: (111)PZT/(111)Pt/(0001) sapphire and [1$\bar{1}$0]PZT/[1$\bar{1}$0] Pt/[10$\bar{1}$0] sapphire. These results suggest that PZT films sputter-deposited on Pt/sapphire possess desirable properties for potential application to pyroelectric devices.

Investigations of Okuyama et al. [49] on PbTi$O_{3}$ films deposited on Pt sheet demonstrate that at substrate temperatures of 300 °C<Ts<450 °C, films have mixed structures of pyrochlore and perovskite types, but those deposited above about 450 °C have the perovskite type (see Table 2 for other processing conditions).

Pignolet et al. [14] studied the structural properties of pure and doped PbTi$O_{3}$ and PZT films deposited on Pt-coated silicon by RF-magnetron sputtering. Depending on the composition, poly films with either a tetragonal or rhombohedral structure were obtained using only a post-deposition thermal treatment (in the absence of any in situ heat treatment). For pure PT, the post-annealing temperature was 800 °C < Tpan < 820 °C, and for PZT films, it was 600 °C<Tpan<650 °C. For pure PT and PZT with less than 40 mol.% zirconium, the structure is tetragonal, while for PZT with more than 50 mol.% zirconium, it is rhombohedral. In between lies the morphotropic phase boundary (MPB), confirming that the composition determines the crystalline structure of the ferroelectric phase. In agreement with observations on films deposited at high substrate temperature [14], the presence of PbO in the target powder has little influence on the film structure except that films sputtered from PbO-enriched targets show smaller concentrations of undesirable phases such as pyrochlore. 

Hetero-epitaxial growth of PT on ST and SrRu$O_{3}$ has been extensively studied. A summary of the results is given below [40]:

1. PT grows in a layer-by-layer mode on TiO_2_-terminated SrTi$O_{3}$ (ST), but this converts to an island-growth mode; after approximately four monolayers of material, this is followed by a layer-by-layer mode if grown on SrRu$O_{3}$ on ST, which is SrO terminated.
2. The island-like growth observed during PT growth on SrRu$O_{3}$ shows a typical “fingerprint” structure, where the islands have a certain height (1 nm) and grow only in lateral size until the islands coalesce. Transmission electron microscopy analysis does not show differences in the interface between PT grown on ST or grown on SrRu$O_{3}$ on ST. With a direct termination switch of SrO-terminated SrRu$O_{3}$ with a monolayer of Ti$O_{2}$, PbTi$O_{3}$ grows layer by layer, as if it were grown on Ti$O_{2}$-terminated ST. Only a difference in relaxation behavior is observed.
3. By contrast, a termination switch of Ti$O_{2}$-terminated ST with one monolayer of SrRu$O_{3}$ does not change the growth behavior as seen on a “thick” layer of SrRu$O_{3}$. PT starts to grow in an island-like growth mode after only three monolayers of SrRu$O_{3}$. This switch while the termination switch after one monolayer of SrRu$O_{3}$ is complete.

Matsubara et al. [19] prepared epitaxial thin films of AB$O_{3}$ perovskite-type oxides, including PT, and (${Pb}_{0.90}{La}_{0.10}$)(${Zr}_{0.65}{Ti}_{0.35}{)}_{0.975}O_{3}$, by RF magnetron sputtering on (100)Si substrate using a buffer epitaxial layer of Mg${A1}_{2}O_{4}$ at 480 °C<Ts<550 °C. PT films composed of a mixture of a and c domains grew in the tetragonal crystal structure, while lead lanthanum zirconium titanate films grew in the cubic structure. The crystallinity of PT film was found to closely correlate to that of the $Mg{Al}_{2}O_{4}$. The a and c axes were aligned with the <100> direction of the underlying Mg${A1}_{2}O_{4}$ lattice, with a preferential orientation controlled by the cooling rate from the processing temperature down to room temperature. 

K. lijima et al. [57] deposited highly c-axis-oriented La-modified PT (PLT) thin films having ${Pb}_{1-x}{La}_{x}{Ti}_{x/4}O_{3}$ compositions with x=0.05 (PLT5), 0.10 (PLT10), and 0.15 (PLT15), on MgO single-crystal and epitaxial Pt thin-film substrates under the conditions of low gas pressure and low deposition rate. For both PT and PL5 samples, α decreases with increasing La content. The α of the PT and PL5 films deposited on MgO is higher than those of the films deposited on the Pt film substrate, presumably because the chemical affinity of MgO to PT and PLT is larger than that of Pt to PT and PL T. The highest α of 0.99, 0.92, and 0.78 were obtained for the films of PL5, PLl0, and PLl5, respectively, which were deposited on MgO single-crystal substrates. The degree of c-axis orientation of the PLT films decreases, and its tetragonality reduces with increasing La content. The results of this study are summarized as follows:

1. Highly c-axis-oriented lanthanum-modified lead titanate (PLT) thin films were obtained by RF-magnetron sputtering under the conditions of low deposition rate and low gas pressure.
2. Upon increasing La content, the degree of c-axis orientation of PLT films decreases, tetragonality c/a decreases, and Curie temperature decreases.

### 5.2. The Effect of (Growth) Substrate Temperature (Ts)

Stringent control on the stoichiometry of oxide composition is of prime importance in the growth of the single-phase perovskite films. A small excess of Pb reduces the film’s melting point and thus improves the crystalline quality of the deposited film. Nevertheless, the discrepancy from the stoichiometric composition generally reduces the crystalline quality of the deposited films. Around 600 °C, the vapor pressure of Pb is excessively high. As a result, the films tend to become Pb deficient. The temperature required to obtain the ferroelectric structure depends on the chemical composition of the film. Highly c-axis-oriented thin films have been obtained with low growth rates and Ts>600 °C [60].

Substrate temperature plays an important role in determining crystal phases. PT films deposited without substrate heating are amorphous but become polycrystalline after four hours of annealing at 700 °C in an oxygen flow of 100 cm^3^/min. The crystal structure is very sensitive to in situ heat treatment. An increase in the *Ts* increases the surface mobility of adatoms, when Ts<200 °C, the PT does not contain any Pb crystallite. For 200 °C<Ts<500 °C the films are composed of a mixture of the ${Pb}_{2}{Ti}_{2}O_{6}$ phase with a pyrochlore structure and the PbTi$O_{3}$ phase with a perovskite structure. For 530 °C < *Ts* < 560 °C, the films show a single phase with the desired perovskite structure. While for Ts>580 °C, the pyrochlore Pb${Ti}_{3}O_{7}$ phase coexists with the PbTi$O_{3}$ perovskite phase [63]. Growth temperature affects both the quality and the growth rate of the growing epitaxial layer. The films on in situ heated substrates can grow epitaxially. As the deposition temperature is increased, the mobility of the species arriving at the film’s surface is increased. Hence growth rate as well as the crystalline quality of the film being deposited is improved. Aside from *Ts*, the film’s crystallization temperature, Tcrys, as well as epitaxial temperature, Tepi, are important factors affecting film morphology. Depending on processing temperature of the perovskite thin films, one of the three phases outlined in Table 7 is obtained as by Wasa et al. [62].

Empirically, for most perovskite oxides ~500 °C<Ts<700 °C. For some cases, different crystal phases will appear at Ts<Tcrys, e.g., for the PT, the pyrochlore phase (${Pb}_{2}{Ti}_{2}O_{7}$) will emerge. Poly phase is achieved either by processing at Ts>Tcrys or by depositing an amorphous film followed by the post-annealing at Ts>Tcrys. Thin films of a single crystalline phase are obtained using a single crystal substrate at Ts>Tepi, due to a vapor phase epitaxy. The amorphous and/or polycrystalline phase are converted into the single crystalline phase by the post-annealing at Tpan>Tepi, as discussed earlier.

The substrate surface temperature itself is governed by the substrate surface conditions such as the density and the atomic spatial distribution and may be controlled by the irradiation of highly energetic particles and/or laser beam onto the growing film surface. It has experimentally been confirmed that *Ts*, is reduced to ~500 °C if, during the co-evaporation of Pb and Ti, the growing film surface is irradiated by oxygen ions. Additionally, superimposing an excimer laser irradiation, further reduces the *Ts* ~ 100 °C to 300 °C, although laser irradiation itself arguably raises the temperature at the film surface [86]. The following parameters influence *Ts*:

1. The kinetic energy of the incident energetic plasma species irradiated on the substrate surface. The surface mobility of the ad-atoms is improved when the kinetic energy of the incident ad-atoms and/or the other energetic plasma species irradiated on the growing film surface is increased. This leads to an effective reduction of *Ts* [87].
2. The incident angle of the irradiated species. This angle defines the amount of energy and/or momentum transferred to the species in the growing film.
3. Tepi is reduced by the reduction of the film growth rate.

S. NAM et al. [65] prepared single-phase perovskite, PZT (${Pb}_{1.1}{(Zr}_{0.4}{Ti}_{0.6}$)$O_{3}$) films by magnetron sputtering at low RF power (100 W), 620 °C on (001) MgO, using PZT ceramic targets incorporating 10 mol.% PbO (see Table 2). The atomic ratio of Zr/Ti was 44/56 in which the Ti content is slightly smaller than that in the targets (40/60). The rocking curve of these PZT films featured FWHM=1.53° for (001) planes. SEM micrography revealed a rough surface with pinholes resulting from the re-evaporation of Pb and re-sputtering of the growing film. Repeating the same experiment with 200 W RF power and 10 (as well as 20) mol.% excess PbO results in a mixed phase containing perovskite, pyrochlore as well as other Pb-deficient phases such as (Zr, Ti)$O_{2}$ and Pb (Zr,${Ti)}_{3}O_{7}$ phases in the [001] direction. These results show that Pb is selectively sputtered from the targets and Pb is severely re-sputtered or re-evaporated from the growing films owing to the high temperature (620 °C) and high RF power (200 W).

### 5.3. The Effect of Processing Gas

The processing gas composition, total pressure, and the partial pressure of oxygen affect morphology stoichiometry, as well as the deposition rate of the growing film.

1. The effect of processing gas composition

Jaber et al. [58] studied the effect of the processing gas composition on the stoichiometry of the growing film. See Table 2. They found that the pyrochlore phase prevails when pure ${Ar}_{2}$ is chosen as the processing gas. Ramesh et al. [88] reported on the effect of the composition of the gas mixture as well as processing pressure on the morphology and characteristics of PT films grown by magnetron sputtering. When gas pressure is varied from 12 to 24 mTorr, the deposition rate is increased, but the preferential orientation (α), defined as the ratio of the integrated intensities of (100) and (001) peaks of X-ray diffraction patterns, is decreased. Below 500 °C, the films were composed of a mixture of pyrochlore and perovskite, above 550 °C, a stable perovskite phase was obtained, while above 650 °C, a second pyrochlore phase ${PbTi}_{3}O_{7}$ co-existed with perovskite structure. This is attributed to the evaporation of Pb from already deposited film due to its high vapor pressure.

R. Thomas [74] prepared PZT thin films on Pt/Ti/Corning 7059 glass substrates by RF magnetron sputtering using a ${Pb(Zr}_{0.5}{Ti}_{0.5})O_{3}$ ceramic target and achieved excellent ferroelectric properties when the deposited amorphous films, were annealed at 650 °C using a chamber pressure of 10 mTorr and oxygen mixing ratio of 50% for 2 h. It was found that the as-deposited amorphous films go through an intermediate pyrochlore phase before crystallizing in the perovskite rosette structure phase, with a 10% deviation from the target stoichiometry (Pb/(Zr+Ti) ≃ 1.1).

2. The oxygen partial pressure in the gas mixture

Most perovskite oxides require some “optimum” oxygen content to achieve certain properties. In particular, the composition of lead-based perovskite thin films depends on oxygen partial pressure during the sputtering process. However, the oxygen partial pressure affects the film’s morphology and the deposition rate.

3. The effect of oxygen partial pressure in the gas mixture on the film’s morphology

The oxygen in the gas mixture reduces the surface mobility of adatoms, by chemically bonding with such atoms and perhaps with the surface atoms, a phenomenon that encourages nucleation and island growth. If oxygen is introduced to the gas mixture, α improves [58]. 

**Figure 23:**
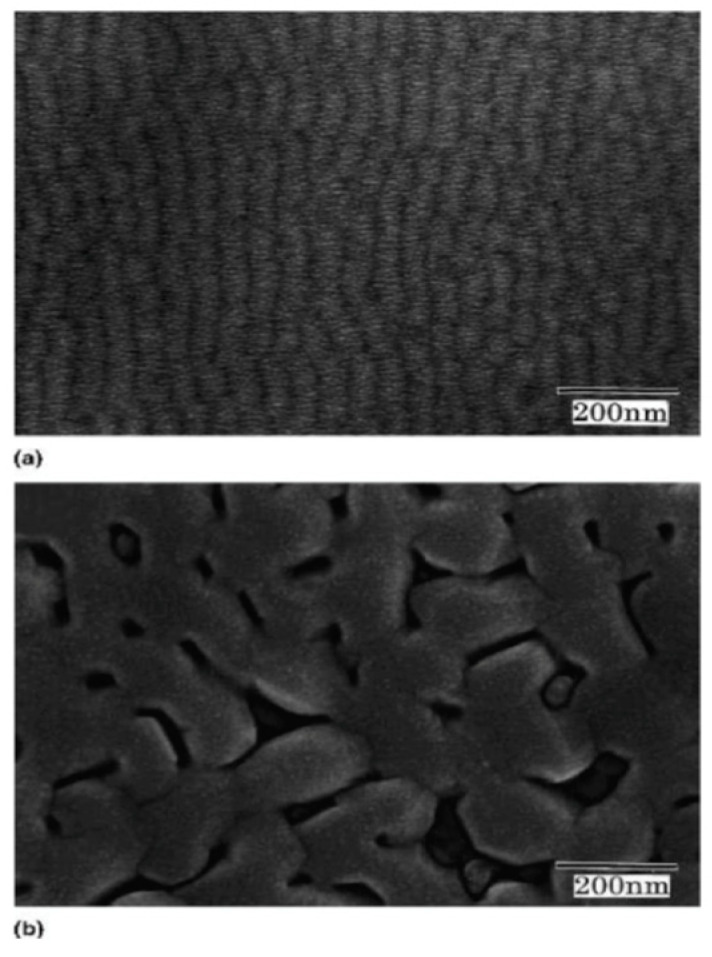
Surface SEM image of PT thin films on miscut (001) ST sputtered at: (**a**) at low partial oxygen pressure, $\left[{Ar}_{2}\right]/\left[O_{2}\right]=20/1$, and (**b**) at high oxygen partial pressure $\left[{Ar}_{2}\right]/\left[O_{2}\right]=20/1$ (the PT film thickness, 130 nm; miscut angle, 1.7°) [89]. Springer Nature grants permission to reproduce material from “Ichikawa, Y.; Matsunaga, T.; Hassan, M.; Kanno, I.;Suzuki, T.; Wasa, K. Growth and structure of heteroepitaxial lead ti-tanate thin films constrained by miscut strontium titanate substrates. Journal of materials research, 2006, 21, pp. 1261–1268. https://doi.org/10.1557/jmr.2006.0162 Page: 1262 Figure 1”.

Ichikawa et al. [89] grew a domain-free/single-crystal (001) PT thin films hetero-epitaxially on 10° miscut (001) ST substrates with various $O_{2}$ partial pressures by RF magnetron sputtering. They describe the structure and growth model of the laterally grown PT thin films in the PT/ST heterostructure and present their analytical results of the temperature variation of lattice parameters based on a plane stress thermo-elastic deformation model. Their magnetron sputtering process conditions are summarized in Table 2. It is found that an increase in oxygen partial pressure, reduces the crystalline quality of the growing PT thin films, although these films attain a single crystal with (001) orientation. Under low oxygen partial pressures ($\left[{Ar}_{2}\right]/\left[O_{2}\right]\geq 20/5$) the film growth is governed by a step flow and results in a continuous film structure with a relatively flat surface as seen in Figure 23a. Under higher oxygen partial pressures ($\left[{Ar}_{2}\right]/\left[O_{2}\right]\leq 20/5$) a hollow (i.e., containing micro-pinholes) three-dimensional film structure stemming from the coalescence of the islands is obtained, as depicted in Figure 23b. From Figure 23, it appears that there exists a critical oxygen partial pressure above which the step-flow growth will turn into a three-dimensional island.

4. The effect of oxygen total pressure on the film deposition rate;

At higher processing pressures, extra ions are produced and hence more ionizing collisions occur within the plasma. This increases the sputtering rate. The collisions, however, decrease the mean kinetic energy of the sputtered species arriving at the substrate. As a result, the mobility of the species depositing on the substrate surface is reduced. The high sputtering (thus deposition) rate in conjunction with the low mobility of the species arriving at the substrate surface tends to encourage 3-D, Volmer–Weber, or mixed mode, SK, rather than the layer-by-layer epitaxial growth of the film and may be verified from Figure 1. Note that at any given temperature, if the growth rate is increased, say by increasing chamber pressure, a poly rather than a single crystalline film can result. This happens because when the total pressure $P_{T}$ (${=P}_{O_{2}}$ + $P_{{Ar}_{2}}$, with $P_{O_{2}}$ and $P_{{Ar}_{2}}$, being the partial pressures of $O_{2}\;and\;{Ar}_{2}$, respectively) is high, above a critical value (Pcr), sputtered species are thermalized within the plasma before arriving at the substrate surface, where they tend to diffuse on the terrace to find energetically suitable positions such as kinks or steps to lean on. If adatoms lose much of their energy by thermalization, they would not diffuse far enough on the terrace to find a suitable position to lean on and thus contribute to layer-by-layer growth. Hence, the adatoms nucleate on the substrate surface and initiate island growth. On the other hand, when $P_{T}<$ Pcr, the adatoms can diffuse far enough on the terrace and facilitate step-flow lateral growth. At Ts=600 °C, when $20/5<[{Ar}_{2}]/[O_{2}]<20/1$, Pcr=0.5 Pa [89]. 

**Figure 24:**
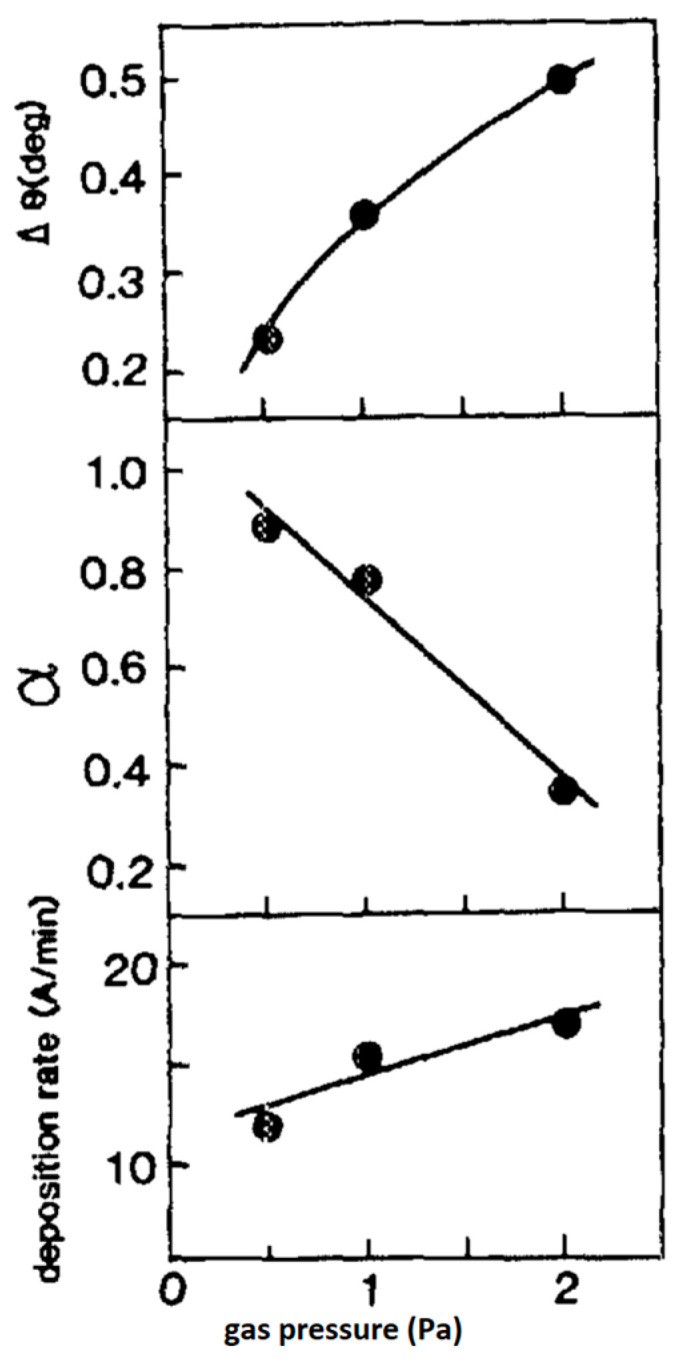
The relationship between the P and v, α as well as Δθ of (001) reflection, for the PLTl0 film [57]. “Copyright © Iijima, K.; Takayama, R.; Tomita, Y.; Ueda, I. Epitaxial growth and the crystallographic, dielectric, and pyroelectric properties of lanthanum-modified lead titanate thin films. Journal of Applied Physics, 1986, 60(8), pp. 2914–2919. https://doi.org/10.1063/1.337078. Journal of Applied Physics. Reproduced by permission of IOP Publishing Ltd. All rights reserved”.

Using an RF-magnetron sputtering method, K. lijima et al. [57] deposited highly c-axis-oriented La-modified PT (PLT) thin films having ${Pb}_{1-x}{La}_{x}{Ti}_{x/4}O_{3}$ compositions with x=0.05 (PLT5), 0.10 (PLT10), and 0.15 (PLT15), on MgO single-crystal and epitaxial Pt thin-film substrates under the conditions of low gas pressure and low deposition rate (see Table 2a). As shown in Figure 24, when P increases, the α decreases and Δθ increases. Jaber et al. [58] studied the effect of the processing gas composition on the deposition rate of the growing film. The deposition rate is found to decrease significantly as the oxygen partial pressure is increased.

5. The effect of the processing gas total pressure on film’s morphology.

Jaber et al. [58] studied the effect of processing gas pressure with targets having X=0.54 at gas pressures of 56 mT and 100 mT. The substrate temperature was fixed at 550 °C. The PT films acquired a highly preferred (111) orientation at 50 mT, while at 100 mTorr, a polycrystalline structure was obtained without any preferred orientation.

### 5.4. The Effect of RF Input Power on the Film’s Morphology

Experimentally it has been verified that RF input power density affects film stoichiometry and the growth rate of PT as well as PZT thin films. K. Torii et al. [90] found that the Pb content in the RF-magnetron sputtered film is proportional to RF power and thereby suggested the use of this tool as a means of controlling the film composition precisely. The use of bias sputtering during the deposition of perovskite films is a very effective means for improving the film quality when the RF power is high since it provides a high deposition rate through the suppression of the damage from the highly energetic oxygen negative ion bombardments. 

**Figure 25:**
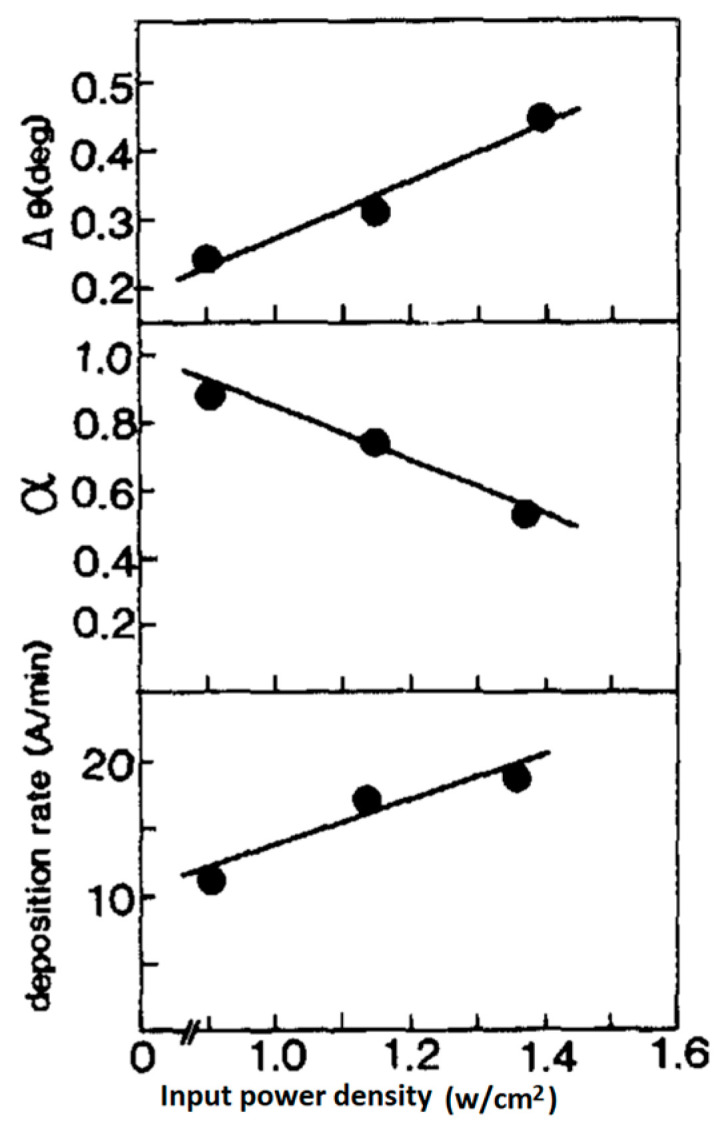
Relationship between the input power and v, α as well as Δθ of (001) reflection, for the PLTl0 film, K. Iijima et al. [57]. “Copyright © Iijima, K.; Takayama, R.; Tomita, Y.; Ueda, I. Epitaxial growth and the crystallographic, dielectric, and pyroelectric properties of lanthanum-modified lead titanate thin films. Journal of Applied Physics, 1986, 60(8), pp. 2914–2919. https://doi.org/10.1063/1.337078. Page: 2915 Figure 2 and Figure 3. Journal of Applied Physics. Reproduced by permission of IOP Publishing Ltd. All rights reserved.”.

Small v(T) induces the formation of the 2nd phase comprising different compounds including pyrochlore, Pb$O_{x}$, and/or Ti$O_{x}$. The formation of the 2nd phase will result from the off-stoichiometry and/or non-uniformity of the film composition. High-quality Pb-based perovskite films require a high deposition rate to minimize the re-evaporation of Pb during the deposition process. Even though the sputtering process is categorized as a high deposition rate process compared with other deposition technologies cited in Table 1, its deposition rate is still low when the growth of micrometer-thick Pb-based perovskite film is of interest. This is because the oxide target in a sputtering system limits the RF power available to plasma. Nevertheless, the growth rate of Pb-based perovskite films can easily be increased by increasing the input RF power. Hayashi et al. [86] reported that the growth rate linearly increases with increasing input RF power density. However, sputtering at high RF power leads to the re-sputtering by energetic negative oxygen ions, which deviates the film composition from stoichiometry and leads to rough surface morphology and target degradation [65]. They find that the α of the film is strongly affected by sputtering conditions. In the case of PLTl0 films, for example, Figure 25 shows the relationships between deposition rate, the degree of c-axis orientation, α, half-width, Δθ of (001) reflection, and the RF input power density. The α decreases and Δθ increases when the input power density increases. Also, Ramesh et al. [88] reported that an increase in power density from 2 to 4 W/cm^2^ adversely affects mismatch due to $\alpha_{T}$. 

### 5.5. The Effect of Applied Negative DC Bias

In addition to RF input power, sometimes a negative DC bias voltage is also applied to the substrate during the sputter deposition of PT film to mitigate the adverse effect of negative oxygen ion bombardment on the substrate surface. This technique also called “bias sputtering” is very effective in improving the quality of the films when the RF power is high and facilitates achieving a high deposition rate through the suppression of the damage anticipated from the highly energetic oxygen-negative ion bombardments.

**Figure 26:**
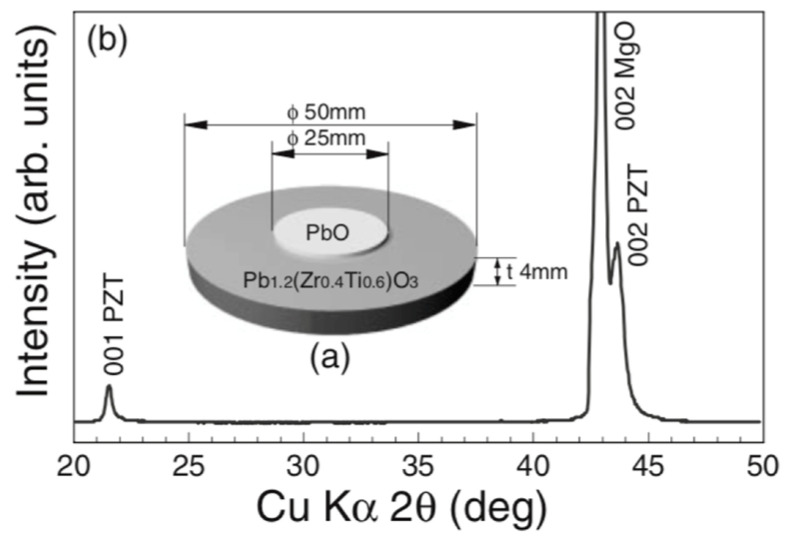
(**a**) schematic diagram illustrating the composite target and (**b**) XRD patterns of the PZT film on a (001) MgO substrate deposited at 200 W using ${(Pb}_{1.2}{Zr}_{0.4}{Ti}_{0.6})O_{3}$ + PbO composite ceramic target [65]. “Copyright © Nam, S. M.; Tsurumi, T. In Situ Epitaxial Growth of Lead Zirconate Titanate Films by Bias Sputtering at High RF Power. Jpn. J. Appl. Phys., 2004, 43(5A), pp. 2672–2676. https://doi.org/10.1143/JJAP.43.2672. Page: 2674 Figure 4. Japanese Journal of Applied Physics. Reproduced by permission of IOP Publishing Ltd. All rights reserved”.

In order to obtain high-quality PZT thin films with high growth rates, Okuyama et al. [55] utilized a negative DC bias voltage. They report on achieving smooth and highly oriented films with improved dielectric properties by increasing the negative bias voltage. S. NAM et al. [65] used a composite target made of PZT ceramics, which had PbO pellets in its center (see Figure 26), to provide the needed constant supply of the Pb and investigated the effect of applying a DC bias to the substrate, on the PZT thin films prepared on (001) MgO single crystal, during sputtering with high RF power.

**Figure 27:**
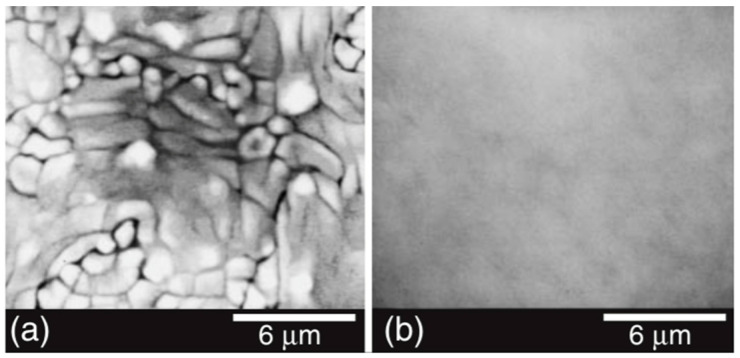
FE-SEM images showing the surface morphology of PZT films deposited on (001) MgO substrates at 200 W under (**a**) 0 V bias voltage and (**b**) −100 V bias voltage using the composite ceramic target [65]. “Copyright © Nam, S. M.; Tsurumi, T. In Situ Epitaxial Growth of Lead Zirconate Titanate Films by Bias Sputtering at High RF Power. Jpn. J. Appl. Phys., 2004, 43(5A), pp. 2672–2676. https://doi.org/10.1143/JJAP.43.2672. Page: 2676 Figure 10. Japanese Journal of Applied Physics. Reproduced by permission of IOP Publishing Ltd. All rights reserved”.

They found that:

1. As the DC bias voltage to substrates increases in either negative or positive voltages, the Ti content and the thickness of PZT films both increase, compared with those prepared without applying DC bias voltage. However, using the negative bias voltage, the chemical composition of the PZT film comes closer to that of the composite target.
2. Despite the redundant Pb content of the composite targets, the composition of Pb in PZT film is self-controlled.
3. Epitaxial PZT films with excellent crystallinity are grown by applying 100 V bias voltage to substrates.
4. As shown in Figure 27, clean, smooth PZT films without voids and pinholes are easily obtained.

For the PZT films with improved morphology, an optimum negative bias voltage exists, which suppresses the bombardments of the energetic negative oxygen ions on the growing film and helps low-energy positive ions to assist in crystallization. Secondary ion bombardment (either external or inherent) is another factor influencing epitaxial growth during deposition. For multi-component oxides, additional parameters come into effect. These include: the stability of various phases of the different oxide components, the mobility of different film species on the substrate, and the nature of the deposition process.

### 5.6. The Effect of the Growing Film’s Thickness (t)

Hetero-epitaxial growth of the thin films involves epitaxial and/or thermal strain due to the lattice parameters and/or thermal expansion coefficient mismatching between the thin films and the substrates. The microstructure of the heteroepitaxial thin films is varied with the film thickness. The heteroepitaxial films show strained structure at a film thickness (t) less than the critical film thickness ($t_{cr}$), where the interface is coherent. For t $>t_{cr}$, dislocations are formed to relax the strain in the interfacial layer, between the film and the substrate. The resultant hetero-epitaxial thin films are referred to as a relaxed structure [91]. In this work, 100<t<300 nm and $200<t_{cr}<250$ nm. The thin films were laterally grown via step-flow growth mode. The PT thin films showed continuous structure without dislocated layers and/or c/a/c/a domains.

### 5.7. The Effect of the Cooling Rate

Matsubara et al. [19] prepared epitaxial thin films of AB$O_{3}$ perovskite-type oxides, including PT, and (${Pb}_{0.90}{La}_{0.10}$)(${Zr}_{0.65}{Ti}_{0.35}{)}_{0.975}O_{3}$, by RF magnetron sputtering on (100)Si substrate using a buffer epitaxial layer of Mg${A1}_{2}O_{4}$ at 480 °C<Ts<550 °C. 

PT films are found to be composed of c-axis and a-axis domains. The intensity of the (001) peak in the X-ray diffraction of the PT film increased with cooling rates. The preferred orientation of the tetragonal PT film, that is c to a domain volume ratio, could be controlled by the cooling conditions after the deposition took place. Highly c-axis oriented films, consisting of more than 90% c domains, were produced by cooling the sample at a high cooling rate, typically 30 °C/min, and by maintaining an RF plasma during cooling a-axis domain prevailed in the samples cooled at 1 °C/min. 

They explain the mechanism of the preferred orientation of the PT film by a balance of compressive stress due to a self-bias effect and tensile stress owing to the lattice mismatch and lattice anisotropy of tetragonal PT. This method is amenable to IC processing.

### 5.8. The Effect of the Excess PbO Content in the Source

Pignolet et al. [63] remark that although the addition of PbO to the PT powder target does not modify the structure of the films, it encourages the formation of the perovskite-type structure at lower substrate temperatures and over a wider range (typically 500–550 °C instead of 530–560 °C). Furthermore, annealed films sputtered from a PbO-enriched target show a smaller concentration of the undesired pyrochlore phases.

## 6. Future Topics

The challenges associated with magnetron-sputtered lead titanate thin films for pyroelectric applications can involve various aspects related to the deposition process, material properties, and application requirements [9,14,19,20,21,22,56,57,58,64,69]. Firstly, achieving epitaxial growth, where the crystal structure of the deposited thin film aligns with that of the substrate, can be challenging. Epitaxy is crucial for maintaining the desired crystalline orientation and enhancing the pyroelectric properties. Secondly, excessive surface roughness can affect the pyroelectric response and compromise the quality of the thin film. Achieving a smooth and uniform surface is essential for optimal performance. Lastly, integrating lead titanate thin films into practical pyroelectric devices presents challenges. Compatibility with device structures and ensuring that the thin films meet specific application requirements can be complex.

## Figures and Tables

**Table 1:** (**a**). Process parameters commonly utilized for in-situ magnetron sputtered of epitaxially grown members of PT family. (**b**). Additional keynotes on the in situ magnetron sputtering of epitaxially grown members of the PT family.

(a)									
Target Diameter, in inch or mm [Target Composition (Form) + wt% or mol.%PbO]	Sputter-Deposition Processing Parameters						Substrate Material (Orientation) (*D_st_*, mm)		Reference
	Gas Mixture		*P*	*Ts*	υ	RF (DC) Power Density or Power			
	${Ar}_{2}$ (%)	$O_{2}$ (%)	Pa or mTorr	°C	nm/min				
[Pb_0.85_ La_0.15_ Ti_0.96_O_3_ + 20 mol.% pbO](P)	90 10	0–90	0.5 Pa	425–475	4–9	RF 1.3 W/cm^2^			[3]
75 mm [Pb_l − x_La _x_Ti _1−x/4_O_3_ + 10 mol.% PbO] (p) x=0.05, 0.10, 0.15.	90	10	1 Pa	600	2.5–3	2 W/cm^2^	Pt/MgO or MgO (80 mm)		[17]
[PT + 5 wt% PbO] (p)	100 50	0 50	12–24 mTorr	300–650	N/A	RF 2–4 W/cm^2^	(70 mm)		[59]
[PT + 5 wt% PbO] (p)	90	10	12–24 mTorr	300	N/A	RF 2–4 W/cm^2^	MgO (70 mm)		[59]
[PT + 5 wt% PbO] (p)	90	10	12–24 mTorr	NA	N/A	RF 2–4 W/cm^2^	Poly-Pt (70 mm)		[59]
80 mm [PT + 20 wt% PbO] (p)	90	10	27 Pa	350–500	3–4	RF 3 W/cm^2^	-Pt sheet-Si(wafer) Pt/Si(wafer)-Pt/mica (35–40 mm)		[49]
80 mm [(TiO_2_ + 10 W% excess Pb_3_O_4_] (p)	90	10	200 mTorr	350–550	3	150 W	-Pt/si(wafer)-Pt/mica (35–40 mm)		[49]
1′ [xPbO + TiO_2_] (P) x=0.54, 0.8, 1	100	0	50–100 mTorr	550–600	N/A	RF 2.6 W/cm^2^	Al_2_O_3_ (0001) (35 mm)		[56]
0.8PbO + 1TiO_2_	N/A	N/A	N/A	600–650	N/A	N/A	SrTiO_3_		[57]
0.54PbO + 1TiO_2_	N/A	N/A	N/A	550–600	N/A	N/A	SrTiO_3_		[57]
1PbO + 1TiO_2_	N/A	N/A	N/A	650–680	N/A	N/A	SrTiO_3_		[57]
75 mm [Pb_1−x_ La_x_ Ti_1−x/4_ O_3_ + 10 mol.% PbO] (P)	50	50	1 Pa	600	1–2	2 W/cm^2^	MgO coated with (100) Pt (80 mm)		[57]
75 mm [Pb_1−x_ La_x_ Ti_x/4_ O_3_ + 10 mol.% PbO] (p) x=0.05, 0.10, 0.15	90	10	1 Pa	550–600	1–2	RF 0.9–1.4 W/cm^2^	MgO/(100) Pt (80 mm)		[57]
1′ [xPbO + TiO_2_] (p) x=0.54, 0.8, 1	100	0	50–100 mTorr	550–600	N/A	RF 2.6 W/cm^2^	Al_2_O_3_ (0001) (35 mm)		[58]
[PbTiO_3_ + 5 wt% PbO] (p)	100 50	0 50	12–24 mTorr	600	1.5	2–4 W/cm^2^	(100) MgO (70 mm)		[59]
[PT + 5 wt% PbO] (p)	90	10	12–24 mTorr	500–650	N/A	2–4 W/cm^2^	MgO (70)		[60]
[Pb (Zr_0.9_ Ti_0.10_ O_3_) + 20 wt% PbO] (p)	80	20	20 mTorr	N/A	N/A	RF 150 W	[111] Pt		[60]
Pb_0.9_ La_0.1_ Ti_0.975_ O_3_	90	10	1 Pa	600	3–4	2 W/cm^2^	(100) Pt/MgO (80 mm)		[61]
Pb-Ti Composite	50	50	0.6 mTorr	150–300	3–60	N/A	NA		[61]
Perovskite compounds, metal composite, multi-target	50	50	5 mTorr	Liq.N_2_ –700	5–7	DC RF	Glass, Sapphire, MgO, SrTiO_3_		[62]
90 mm [PT] (P)	100	0	1 Pa	500–650	4–6	3 W/cm^2^	9×9×0.36 mm (100) Si or Pt on (100) Si or Si_3_Ti_5_ on (100) Si (60 mm)		[63]
80 mm [PT+ excess Pb_3_O_4_]	90	10	27 Pa	350–550	3–4	150 W	-Pt sheet-Si(wafer) -Pt/Si(wafer)-Pt/mica (35–40 mm)		[63]
100 mm [PT] (P, Sintered, 99.9%)	90	10	4 Pa	630	N/A	75 W	patterned Pt on SrTiO_3_		[63]
3′ [PbLaZrTiO_3_]	10	4	0.53 Pa	476	60	Pb: 0.69 W/cm^2^ Ti: 3.67 W/cm^2^	Pt/Ti/SiO_2_/Si		[63]
[Pb (Zr_0.9_Ti_0.1_) O_3_ + 20 wt% PbO] (P)	80	20	20 mTorr	610	N/A	150 W	(111) Pt		[64]
Pb_x_ (Zr_0.4_Ti_0.6_) O_3_ Ceramics: x=1.1, 1.2 Composite ceramics: x=1.2	80	20	20 mTorr	620	5	100 W, 200 W RF	(001) MgO single crystal (30)		[65]
60 mm [(Pb, Ti) wafers]	67	33	3 Pa	700	N/A	DC Pb: 0.32 W/cm^2^ Ti: 6.37 W/cm^2^	(100 mm)		[66]
100 mm [PbTiO_3_] (P)	N/A	N/A	N/A	600	3–5	N/A	(001) ST, miscut: 0.9°–10° (30 mm)		[67]
(**b**)									
**Notes**								**Reference**	
Subs = Pt/Ti/Glass, Target was Pb (Zr, Ti) O_3_ ceramic disk (dia =100 mm), 100% pre-sputtering duration =15 min								[7]	
It used HVAC, base pressure = 10^−6^ Torr; substrate: cleaved MgO (100) annealed at 1200 °C for 12 h, in air.								[14]	
(100) Pt electrodes (0.2 μm thickness) deposited on the (100) cleaved and polished. MgO single crystals, or on the (100) cleaved and polished MgO substrates.								[17]	
The target pressed on a quartz plate.								[49]	
Sputtering target consisted of cold-pressed mixed oxide powders of PbO and TiO_2_ (purity 99.9%) with composition: X PbO+Y TiO_2_, where X and Y represent the mole concentration of PbO and TiO_2_, respectively.								[56]	
Subs = Pt/Ti/SiO_2_/Si 100 mTorr, ambient, water-cooled, substrate Al_2_O_3_ [0001] at 700 °C.								[57]	
It used the bias sputtering.								[68]	
It used the UHV system with a base pressure of 10^−6^ Torr, (100) Cleaved MgO crystals were annealed for 3 h at 1200 C in air. Introducing O_2_ to processing gas reduced deposition rate. Optimum O_2_ content for stoichiometric PT is approximately 10%. Amorphous films were obtained for the substrate temperature below 400 °C. Perovskite films were obtained above 500 °C. The epitaxial films that were highly c−axis oriented along the substrate surface normal were obtained above 600 °C. Their epitaxial relation was PbTiO_3_ (100)/MgO (100).								[60]	
Processing parameters for correct stoichiometry.								[63]	
To prepare [111]-oriented epitaxial PZT films, [111] oriented Pt films with a thickness of about 0.3 μm were deposited epitaxially on (0001) sapphire by RF magnetron sputtering at 450 °C.								[64]	
Deposition time was 30 min.								[65]	
It used the multi-target sputtering technique.								[66]	
The situ effects of platinized silicon substrates on the properties of PZT thin films by magnetron sputtering.								[69]	
Used cooling rate = 100°/min, in air.								[67]	
Used bias voltage to substrate: −200 V, −100 V, −50 V, 0 V, +50 V, +50 V, +100 V.								[40]	

**Table 2:** (**a**). Magnetron sputtering parameters commonly utilized in preparation of PT family films prepared by solid-state crystallization (preparation of the amorphous film). (**b**). Post-annealing parameters utilized in preparation of PT family films by solid-state crystallization of the amorphous films, outline in Table 2(a).

(a)								
Target Diameter, in inch or mm [Target Composition (Form) + w% or mol.%PbO]	Sputter-Deposition Processing Parameters						Substrate Material & (SUBSTRATE to Target Distance)	Reference
	Gas Mixture (Ratio or %)		Pressure	*Ts*	υ	RF (DC) Pw (W) or Pwd (W/cm^2^)		
	${Ar}_{2}$ (%)	$O_{2}$ (%)	Pa or mTorr	(°C)	$\left(nm/min\right)$			
2.75′ [PbZr_0.52_Ti_0.48_ O_3_] 0.9(P) + [PbO] 0.1(P)	9	1	2.7 Pa	250	2.66	40 W	Pt/Ti/SiO_2_/Si (N/A)	[21]
90 mm [PT + 10 w%? PbO] (P)	100	0	0.8–1 Pa	N/A	3–10	100–150 W	Pt/Si (60 mm)	[14]
90 mm [PbZrO_3_ (P) + 10 w% PbO (P)]	100	0	N/A	N/A	N/A	N/A	N/A	[14]
[PT + 5 w% PbO] (P)	100 50	50 50	12–24 mTorr	300	N/A	2–4 W/cm^2^	Pt (70 mm)	[60]
[Pb_3_O_4_ + TiO_2_] (P)	90	10	200 mTorr	350–450	N/A	N/A	3 × 3 mm Si, SiO_2_/Si, Pt/SiO_2_/Si	[72]
90 mm [PbZrO_3_, PT mixture + 10 w% PbO] (P), near MPB PbZr_0.53_ Ti_0.47_O_3_	100	N/A	0.6–0.8	N/A	8–10	100–150 W	Synthetic Al_2_O_3_ or (100) Si covered with (111) Pt	[73]
(**b**)								
**Target Diameter,** **in** inch **or** mm **[Target Composition (Form) + w% or mol.%PbO]**	**Post Annealing**						**Substrate Material** **& Substrate** **Target** **Distance (mm)**	**Reference**
	**Tpan (** °C **) & Time (min)**			**Medium Gas (SCCM)**				
2.75′ [PbZr_0.52_Ti_0.48_ O_3_] 0.9(P) + [PbO] 0.1(P)	700 & 20			Ar & O_2_			Pt/Ti/SiO_2_/Si (50 mm)	[21]
90 mm [PT + 10 w%PbO] (P)	800–820			O_2_			Pt/Si (60 mm)	[14]
90 mm [PbZrO_3_ (P) + 10 w% PbO (P)]	600–650			O_2_			Pt/Si (60 mm)	[14]
[PT + 5 w% PbO] (P)	475			N/A			MgO (70 mm)	[59]
[PT + 5 w% PbO] (P)	600–650			N/A			Poly-Pt (70 mm)	[60]
[Pb_3_O_4_ + TiO_2_] (P)	CW laser, 6 mm dia, 10–40 W, 1–10 s.			N/A			3 × 3 mm Si, SiO_2_/Si, Pt/SiO_2_/Si	[72]
90 mm [PbZrO_3_, PT mixture + 10 w% PbO] (P), near MPB PbZr_0.53_ Ti_0.47_O_3_	600			O_2_ (10)			Synthetic Al_2_O_3_ or (100) Si covered with (111) Pt	[73]
100 mm [Pb (Zr_0.5_ Ti_0.5_) O_3_] ceramic disc	650			O_2_			Pt/Ti/corning 7059 (50 mm)	[74,75]

**Table 3:** Avrami constants for various phase transformation behavior [76].

Growth Dimension	Depletive Nucleation		Constant Nucleation	
	Interface	Diffusion	Interface	Diffusion
1-Dimensional	1	1/2	2	3/2
2-Dimensional	2	1	3	2
3-Dimensional	3	3/2	4	5/2

**Table 4:** Crystallographic data and the TCE for important substrate materials [80].

Crystalline Material	Crystal System	Structure	Lattice Constant $\left(A{}^{\circ}\right)$	Oxygen Distance $\left(A{}^{\circ}\right)$	CTCE $\left(10^{-6}\right)$ at 25° (/K)	Reference
Sapphire	Trigonal	Hexagonal	a=4.763c=1.3003	2.75	4.5||to a-axis 5.3||to c-axis	[60,81]
SrTi$O_{3}$	Cubic	Perovskite	a=3.905	2.76	9	[81]
PbTi$O_{3}$	Tetragonal	Perovskite	a=3.908c=4.158	2.76 2.93	12 12	[46,82]
PbTi$O_{3}$	Cubic	Perovskite	a=3.961	N/A	12	[46,82]
PLZT	Cubic	Perovskite	a=4.091	N/A	N/A	[40,83]
CaF_2_	Cubic	NaCl	a=5.451	N/A	18.85	
SrF_2_	Cubic	NaCl	a=4.148	N/A	18.4	
MgO	Cubic	NaCl	a=4.212	N/A	13.8	
MgAl_2_$O_{4}$	Cubic	NA	a=8.08	2.86	7.33	

**Table 5:** Lattice constant and O-O spacing for pseudo-cubic structure PbTi$O_{3}$ families [73].

Film Composition	Lattice Constant (A°)	O-O (A°)
PLZT (9/65/35)	4.09	2.89
PLZT (0/0/100):PT	4.02	2.85
PLZT (14/0/100)	3.99	2.82
PLZT (21/0/100)	3.96	2.80
PLZT (28/0/100)	3.95	2.79
PLZT (35/0/100)	3.94	2.79
PLZT (42/0/100)	3.93	2.78

**Table 6:** Mismatch between (111) PZT (90/10), (111) Pt, and (0001) sapphire.

Film/Buffer Layer	Substrate	Mismatch (%)
PZT	Sapphire	6.2
Pt	Sapphire	1.1
PZT	Pt	5

**Table 7:** Phase-processing temperature relationship of the perovskite thin films, Wasa et al. [62].

Perovskite Thin Films	Temperature Relationship	Phase-Processing
Amorphous	Ts<Tcrys	Data
Poly	Ts>Tcrys	
	Ts<Tcrys	Post-annealing
Single crystalline	Ts>TepiTs<Tcrys	(Vapor phase epitaxy) Post-annealing (Solid phase epitaxy)

## Data Availability

Not applicable.
